# Patient-reported outcome measures for fatigue in patients with chronic kidney disease: a systematic review

**DOI:** 10.1136/bmjopen-2025-099592

**Published:** 2025-07-16

**Authors:** Anastasia Hughes, Angela Ju, Rosanna Cazzolli, Martin Howell, Chandana Guha, Adeera Levin, Karine Manera, Armando Teixeira-Pinto, Luca G Torrisi, David C Wheeler, Germaine Wong, Rebecca Wu, Allison Jaure

**Affiliations:** 1 Sydney School of Public Health, The University of Sydney, Sydney, New South Wales, Australia; 2 Centre for Kidney Research, The Children’s Hospital at Westmead, Sydney, New South Wales, Australia; 3 Leeder Centre for Health Policy, Economics and Data, Faculty of Medicine and Health, University of Sydney, Sydney, New South Wales, Australia; 4 Division of Nephrology, The University of British Columbia, Vancouver, British Columbia, Canada; 5 Department of Renal Medicine, University College London, London, UK

**Keywords:** Fatigue, NEPHROLOGY, Quality of Life, Patient Reported Outcome Measures, Chronic renal failure

## Abstract

**Abstract:**

**Objective:**

Fatigue is a common and debilitating symptom that is associated with an increased risk of mortality, dialysis initiation and hospitalisation among patients with chronic kidney disease (CKD). The aim of this study was to identify the characteristics, content and psychometric properties of patient-reported outcome measures (PROMs) used to measure fatigue in patients with CKD not requiring kidney replacement therapy (KRT).

**Design:**

Systematic review. The characteristics, dimensions of fatigue and psychometric properties of these measures were extracted and analysed.

**Data sources:**

We searched MEDLINE, Embase, PsycINFO and CINAHL from database inception to February 2023.

**Eligibility criteria for selecting studies:**

All studies that reported fatigue in patients with CKD stages 1–5 not receiving KRT.

**Results:**

We identified 97 studies (20 (21%) randomised trials, 2 (2%) non-randomised trials and 75 (77%) observational studies). 27 different measures were used to assess fatigue, of which three were author-developed measures. The 36-Item Short Form Health Survey (SF-36) and Kidney Disease Quality of Life – Short Form (KDQOL-SF) were the most frequently used measures (41 (42%) and 24 (25%) studies, respectively). Six (22%) measures were specific to fatigue (Chalder Fatigue Questionnaire, Functional Assessment of Chronic Illness Therapy – Fatigue Scale, Functional Assessment of Cancer Therapy-Fatigue, Fatigue Severity Scale, and author developed Chen & Ku 1998, and Hao *et al* 2021) while 21 (78%) included a fatigue subscale or item within a broader construct for example, quality of life. Various content domains assessed included tiredness, ability to think clearly, level of energy, muscle weakness, ability to concentrate, verbal abilities, motivation, memory, negative emotions and life participation. Only two measures (Chronic Kidney Disease Symptom Index – Sri Lanka, Kidney Symptom Questionnaire) were developed specifically for CKD, but they were not specific to fatigue. Six measures (Chronic Kidney Disease Symptom Index – Sri Lanka, Functional Assessment of Cancer Therapy – Anemia, Revised Illness Perception Questionnaire, Kidney Symptom Questionnaire, Short Form 6 Dimension and 36-Item Short Form Health Survey) had been validated in patients with CKD not requiring KRT.

**Conclusion:**

PROMs used to assess fatigue in patients with CKD vary in content and few were specific to fatigue in patients with CKD not requiring KRT. Data to support the psychometric robustness of PROMs for fatigue in CKD were sparse. A validated and content-relevant measure to assess fatigue in patients with CKD is needed.

**STRENGTHS AND LIMITATIONS**

- Our explicit focus was to assess the characteristics and psychometric properties rather than the effect of interventions; therefore, we did not conduct a risk of bias assessment.
- There may be other measures of fatigue that have not been included in our review that could potentially be appropriate for patients with chronic kidney disease (CKD).
- The CKD focused search strategy may not have identified the full extent of cultural adaptations and translations available for the instrument’s availability.

## Introduction

 Fatigue is a common and debilitating symptom experienced by patients with chronic kidney disease (CKD) not yet requiring kidney replacement therapy (KRT), which is also associated with an increased risk of mortality, dialysis initiation and hospitalisation.^1^ Fatigue contributes to symptom burden and impaired quality of life and life participation.^2^,^5^ The challenges in managing fatigue are real and due to the fact that the causes are multifactorial, and evidence supporting interventions to manage fatigue in patients with CKD is limited.^1 6 7^

Fatigue, broadly defined as a subjective state of exhaustion or tiredness,^8^ has been identified as an important outcome by patients with CKD, caregivers, health professionals and researchers.^2 9^ Yet, fatigue is reported infrequently and inconsistently in trials, with a diverse range of tools used.^1 10^ Consequently, this can limit the reliability and comparability of the evidence for interventions to improve fatigue in patients with CKD.

This study aimed to identify the characteristics, content and psychometric properties of the patient-reported outcome measures (PROMs) used to measure fatigue in patients with CKD not requiring KRT, to inform the identification, development and validation of a psychometrically robust measure for fatigue that is meaningful to patients, caregivers and health professionals.

## Methods

### Selection criteria

We searched for randomised and non-randomised controlled trials, and observational studies that included at least one PROM that assessed fatigue in patients with CKD not requiring KRT. Fatigue was defined as tiredness, muscle weakness and level of energy.^8^ Studies that included adult patients aged 18 years or over with CKD stages 1–5 not requiring KRT (peritoneal dialysis, haemodialysis or kidney transplantation) were eligible. No time or language restrictions were applied. Studies reporting clinician-reported or proxy-reported outcomes for fatigue were excluded. Studies that assessed sleep quality, sleep disturbances and insomnia were excluded as these were regarded as being different outcomes to fatigue. Abstract only citations were included only if they provided sufficient information about the tool used to assess fatigue. The systematic review adheres to the COnsensus-based Standards for the selection of health Measurement INstruments (COSMIN) guidelines for systematic reviews on patient-reported outcome measures.^11^ The Preferred Reporting Items for Systematic reviews and Meta-Analyses checklist is provided in online supplemental table S1.

### Study sources and measures

**Figure 1:**
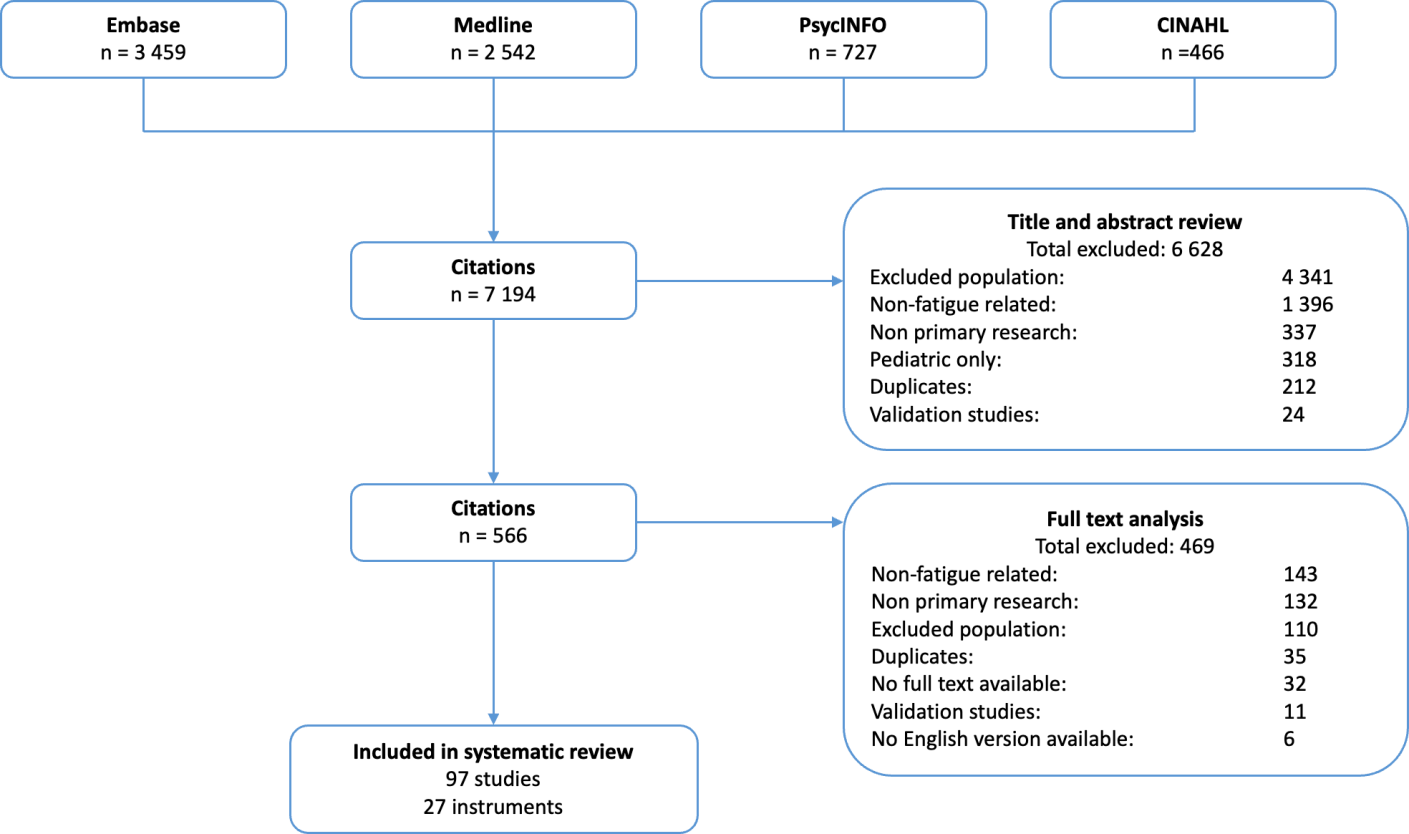
PRISMA flowchart of search and selection process.

We searched MEDLINE, Embase, PsycINFO and Cumulative Index to Nursing and Allied Health Literature (CINAHL) from database inception to February 2023. The search strategy is provided in online supplemental table S2. The initial literature search and screening were conducted by author (AH) and search results were reviewed by author AJa to ensure accuracy. The data extraction was conducted by AH and checked by author AJa. Studies that did not meet the inclusion criteria were excluded (figure 1).

### Data extraction and analysis

From each study, A.H extracted the author, publication, year, sample size (patients with CKD not requiring KRT), country, type of intervention (if applicable) and tool used to assess fatigue. The characteristics of each tool including the response format, number of items, recall period, cost of license, completion time and language were summarised in reference to the study source. Where the completion time was unavailable, it was estimated based on the number and length of items, assuming an average of 12 s per item.^12^ To extract psychometric data for each tool, A.H searched for validation studies in patients with CKD not requiring KRT.

### Dimensions of fatigue

We extracted all items relating to dimensions of fatigue, including fatigue subscales from measures designed to assess multiple or broader concepts (eg, quality of life). The dimensions of fatigue were divided into two groups: measurement and content. Measurement dimensions included severity, frequency, duration and change. Content dimensions included tiredness, muscle weakness, level of energy, impact of fatigue on ability to think clearly, ability to concentrate, verbal abilities, motivation, memory, negative emotions and impact on life participation. Definitions of fatigue are presented in table 1. The dimensions of fatigue were derived from a systematic review of PROMs for fatigue in dialysis.^10 13^ To ensure all dimensions were included, we also examined the Patient-Reported Outcomes Measurement Information System (PROMIS) fatigue measure, no additional dimensions were identified.^14^

**Table 1:** Definitions of the dimensions of fatigue assessed by each PROM

Dimension	Definition	Example of an item
Measurement dimensions		
Severity	The extent of overall fatigue felt by the patient	How severe is the fatigue you have felt?
Frequency	The number of times a patient has felt fatigued within a certain time frame	Over the past 7 days, how often have you felt fatigued?
Duration	The length of time a patient felt fatigued	How long have you felt fatigued?
Change	Differences in fatigue felt by a patient within a certain time frame	To what extent has your fatigue changed within the last 7 days?
Content dimensions		
Tiredness	Desire to rest, feeling exhausted	I feel tired; Do you feel tired? Do you feel the need to rest/stay in a chair/bed all day?
Limb/muscle weaknesses	Physical weakness in specific parts of the body	I have less strength in my muscles; Do you feel able to use your muscles to full capacity?
Level of energy (general energy, physical energy)	Amount of energy available for daily activities and physical mobility	I feel lively; How well are you able to get around?
Ability to think clearly	Impact of fatigue on the ability to think clearly	I feel alert; Are you able to think clearly?
Ability to concentrate	Impact of fatigue on the ability to concentrate	What I am doing something I can’t keep my thoughts on it; Are you able to focus?
Memory	Impact of fatigue on the ability to recall and remember	I have trouble remembering things; How well are you able to recall information when you are fatigued?
Verbal abilities	Impact of fatigue on the ability to speak clearly	I make slips of the tongue when speaking; When fatigued, how clear is your speech?
Motivation	Impact of fatigue on the patient’s desire to engage in activities (eg, social, recreation, leisure, work, daily activities)	I dread having to do things; Are you motivated to engage in activities when fatigue?
Negative emotions	Impact of fatigue on the patient’s emotions (eg, sad, irritable)	I am easily irritated; How is your mood affected when you are fatigued?
Life participation	Impact of fatigue on the patient’s ability to participate in life activities (eg, daily activities, social, recreation, leisure, work	Due to my fatigue, I have to limit my activities; Does your ability to participate in life change when you are fatigued?

Adapted from Ju December 2017.

### Assessment of psychometric properties

As recommended by COnsensus-based Standards for the selection of health Measurement INstruments-Core Outcome Measures in Effectiveness Trials (COSMIN-COMET), we manually searched for the validation studies of PROMs identified in the review reporting psychometric robustness in patients with CKD not requiring KRT. We searched for all validation studies reporting the psychometric robustness of the tool and extracted the psychometric assessment. We extracted and summarised the validity (content, criterion, cross-cultural, known groups, structural) and reliability (responsiveness, test-retest, internal consistency) of the included tools in patients with CKD not requiring KRT. To do this, we followed the COSMIN-COMET framework.^15 16^ Definitions of psychometric properties are provided in online supplemental table S3.

### Public and patient involvement in research

Patients and caregivers are involved in the Standardised Outcomes in Nephrology (SONG) Organising Committee and SONG-CKD Expert Working Group. There are patients and caregivers involved as co-authors in this research. Patients and caregivers will be involved in the dissemination of these results through networks and organisations to advocate for the use of the SONG outcome measures in trials.

## Results

### Characteristics of studies

After screening, we included 97 studies involving 71 039 patients with CKD across 33 countries. 20 (21%) were non-randomised trials, 2 (2%) were randomised trials, and 75 (77%) observational studies. The search results are depicted in figure 1 and the characteristics of the studies are shown in online supplemental table S4.

### Characteristics of measures

Across the 97 studies, 27 tools were used to assess fatigue in patients with CKD not requiring KRT. The most frequently used instruments were the Short Form-36 (SF-36) (41 (42%) studies), followed by the Kidney Disease Quality of Life-Short Form (KDQOL-SF) (24 (25%)). A detailed summary of the characteristics and frequency of tools is provided in table 2.

**Table 2:** Characteristics of PROMs used to assess fatigue in CKD

PROM	Response format	Number of items	Recall	Cost	Completion time†	Specific to fatigue; Specific to CKD*	Language^§^	Frequency of use (number of studies)
15D^33^	5-point ordinal scale	15	Current	No charge	~5 min	No; no	Multiple languages including English, Arabic, Chinese, Danish, Dutch, English, French, German and Portuguese	1
BDI-I^34^	Multiple choice	21	2 weeks	Fee. Contact author	<5 min	No; no	More than 20 languages	1
CFQ^35^	Yes/no, 4-point Likert scale	11	Past 4 weeks	No charge	<3 min	Yes; no	Multiple	1
CKD-SI^17^	Yes/no, If yes, 5-point Likert scale (very mild to very severe)	25	Past 7 days	Permission from author	~5 min	No; yes	English	1
DSI^36^	Yes/no, 5-point Likert scale	30	Past week	Free	~10 min	No; no	Multiple	1
ESS^37^	4-point Likert scale	8	In recent times	License fee for some, others not	2–5 min	No; no	Multiple	5
FACIT-Fatigue^38^	5-point Likert type scale	13	Past 7 days	Non-commercial use assessed per case basis. Licencing fee not typically applied to investigator-led, students and clinical use	<5 min	Yes; no	Multiple	4
FACT-An^39^	5-point Likert style scale	47	Past 7 days	Non-commercial use assessed per case basis. Licencing fee not typically applied to investigator-led, students and clinical use	10–15 min	No; no	Multiple languages including, English, Danish, Spanish, French and Chinese	1
FACT-F	5-point Likert type scale	40	Past 7 days	Non-commercial use assessed per case basis. Licencing fee not typically applied to investigator-led, students and clinical use	10–15 min	Yes; no	Multiple	2
FSS^40^	7-point Likert scale	9	Past 7 days	Free	<2 min	Yes; no	Multiple	1
IPQ-R^41^	Yes/no, 5-point Likert scale	84	Not stated	Not stated	~30 min	No; no	English, Norwegian, Dutch and French	1
KDQOL^42^	Yes/no, 3-/5-/6-point Likert scale	134	Last 30 days	Available on request to those measuring QOL in patients on dialysis	~27 min	No; no‡	Multiple including English, French, Japanese and Spanish	1
KDQOL-36^43^	Yes/no, 3-/5-/6-point Likert scale	36	Current, past 4 weeks	No charge	~10 min	No; no‡	Multiple including English, French, Cantonese Chinese, Korean, Spanish and Turkish	6
KDQOL-SF^44^	Yes/no, 3-/4-/5-/6-point Likert scale	80	Past 4 weeks	Free	~16 min	No; no‡	Multiple	24
KSQ^20^	5-point Likert scale	13	Not stated	Available for clinical or research use under licence. Contact author.	~5 min	No; yes	English	1
LASA^45^	9-point Likert scale	5	Not stated	Not stated	~1 min	No; no	English, German	2
LUSS^20^	5-point Likert scale	11	Not stated	Not stated	<5 min	No; no	English	1
MOS^46^	Not stated	116	Not stated	Not stated	~24 min	No; no	English	1
MOS-Sleep-R^47^	5-point Likert scale	12	Past 7 days, past 4 weeks	Not stated	~2 min	No; no	English	1
PSQI^48^	4-point Likert scale and open-ended questions converted into scaled scores	19	Past month	Not stated	5–10 min	No; no	English	3
QIDS-SR^49^	4-point Likert scale	16	Past 7 days	Free	<4 min	No; no	English and other translations available	1
SF-6D^50^	4-/5-/6-point ordinal scale	6	Current	No charge for non- commercial use. License fee for commercial use	<2 min	No; no	Multiple	2
SF-12^51^	Yes/no, 3-/5-/6-point Likert scale	12	Past 4 weeks	License fee	~2 min	No; no	Multiple	4
SF-36^52^	Yes/no, 3-/5-/6-point Likert scale	36	Past 4 weeks	Annual licence fee	5–10 min	No; no	Multiple	41
Author developed								
Chen and Ku, 1998^53^	5-point Likert scale	25	Data unavailable	Data unavailable	~5 min	Yes; no	Unknown	1
FS developed by Lin 2006^54^	4-point Likert scale	26	Past month	Not stated	~5 min	No; no	Unknown	1
Hao *et al* 2021^55^	Five check boxes—very much/somewhat/a little bit	3	Not stated	Not stated	~1 min	Yes; no	English	1

* CKD not requiring kidney replacement therapy.

† Where time completion data were unavailable, authors estimated based on 12 s per item.

‡ Developed for dialysis and/or transplant.

§ Language availability not necessarily validated in another language.

BDI-I, The Beck Depression Inventory; CFQ, Chalder Fatigue Questionnaire; CKD, chronic kidney disease; 15D, 15 Dimensions; DSI, Dialysis Symptom Index; ESS, Epworth Sleepiness Scale; FACIT-Fatigue, Functional Assessment of Chronic Illness Therapy – Fatigue Scale; FACT-An, Functional Assessment of Cancer Therapy – Anemia; FACT-F, Functional Assessment of Chronic Illness Therapy – Fatigue; FSS, Fatigue Severity Scale; ICECAP-O, ICEpop CAPability measure for Older people; IPQ-R, Revised Illness Perception Questionnaire; KDQOL, Kidney Disease Quality of Life; KDQOL-36, Kidney Disease Quality of Life – 36-Item Questionnaire; KDQOL-SF, Kidney Disease Quality of Life – Short Form; KSQ, Kidney Symptom Questionnaire; LASA, Linear Analog Scale Assessment; LUSS, Leicester Uraemic Symptom Score; MOS, Medical Outcomes Study; MOS-Sleep-R, Medical Outcomes Study Sleep Scale Revised; PSQI, Pittsburgh Sleep Quality Index; QIDS-SR, Quick Inventory of Depressive Symptomatology-Self Report; SF-12, 12-Item Short Form Health Survey; SF-36, 36-Item Short Form Health Survey; SF-6D, Short-Form 6 Dimension.

No tools were designed to specifically assess fatigue in patients with CKD not requiring KRT. Two tools, the Kidney Symptom Questionnaire (KSQ) and Chronic Kidney Disease-Symptom Index (CKD-SI), that were designed to assess broader constructs (quality of life and symptom burden) in patients with CKD not requiring KRT, included fatigue as an item. Six (22%) tools (Chalder Fatigue Questionnaire (CFQ), Functional Assessment of Chronic Illness Therapy – Fatigue (FACIT-Fatigue), Functional Assessment of Cancer Therapy – Fatigue (FACT-F), Fatigue Severity Scale (FSS), and author developed Chen & Ku, and Hao *et al*) were specifically designed to assess fatigue. Six (22%) tools were designed for kidney disease more broadly (either dialysis and/or transplant) (KDQOL, KDQOL-36, KDQOL-SF, Dialysis Symptom Index (DSI), author developed FS by Lin, and Chen & Ku 1998). There were 3-author developed tools (Chen & Ku 1998, FS by Lin, Hao *et al*). 17 tools were used in single studies and 10 tools were used in two or more studies.

The recall period for tools ranged from current to the past month. Most tools asked respondents to recall the past 7 days (8 tools, 30%) or past 4 weeks (9 tools, 33%). The time taken to complete each tool ranged from less than 2 minutes to 30 minutes. The cost of obtaining and using tools ranged from no charge, contact/permission from author and a licensing fee. 12 (44%) tools were free of charge and nine (33%) were unclear about licensing. 18 (67%) tools were available in other languages, in addition to English.

### Content of measures

In total, there were 14 fatigue dimensions (four classified under measurement and 10 under content) identified across all the tools. The number of dimensions included in individual tools ranged from two to seven, with an average of four. 25 (93%) of the tools assessed only one measurement dimension of fatigue, most commonly severity (15, 56%) and frequency (13, 48%). The top five most frequently assessed content dimensions were tiredness (21, 78%), level of energy (16, 59%), muscle weakness (11, 41%), motivation (10, 37%) and negative emotions (8, 30%). The content of the tools is shown in table 3 and online supplemental table S5.

**Table 3:** Dimensions of fatigue assessed by each measure

Measure	Dimensions
15D^33^	Walking (indoors, outdoors), read, sleep, eat, speak, housework, work outside the home, social interactions (friends, family, meetings, recreation, leisure)
BDI-I^34^	Sadness, pessimism, failure, loss of pleasure, guilt, punishment, self-esteem, self-criticism, suicidal ideas, crying, agitation, loss of interest, indecision, devaluation, lack of energy, changes without a sleep pattern, irritability, changes in appetite, difficulty concentrating, tiredness and loss of interest in sex
CFQ^35^	Tiredness, rest, sleepy or drowsy, problems starting things, lack of energy, less strength in muscles, feeling weak, difficulties concentrating, slips of the tongue when speaking, difficulty finding the right word, memory
CKD-SI^17^	Loss of appetite, nausea, vomiting, diarrhoea, lethargy, changes in skin colour, swelling of arms of legs, difficulty in breathing, hiccups, difficulty keeping legs still, numbness/tingling of hands/feet, lack of energy, trouble with memory, weight loss, bone/joint pain, muscle camps
DSI^36^	Constipation, nausea, vomiting, diarrhoea, decreased appetite, muscle cramps, swelling in legs, shortness of breath, light-headedness or dizziness, restless legs and difficulty keeping legs still, numbness or tingling in feet, feeling tired or lack of energy, cough, dry mouth, bone or joint pain, chest pain, headache, muscle soreness, difficulty concentrating, dry skin, itching, worrying, feeling nervous, trouble falling asleep, feeling irritable, feeling sad, feeling anxious, decreased interest in sex, difficulty becoming sexually aroused
ESS^37^	Chance of dozing: sitting and reading, watching tv, sitting inactive in a public space (theatre, meeting), passenger in a car without break, lying down to rest, sitting and talking to someone, sitting quietly after lunch without alcohol, in traffic
FACIT-Fatigue^38^	Fatigued, feeling weak, listless, tired, trouble starting/finishing things, energy, able to do usual activities, sleep during the day, too tired to eat, frustrated being too tried to do usual activities, limited social activity due to tiredness
FACT-An^39^	Lack of energy, nausea, pain, treatment, feeling ill, time in bed, friends and family, sex life, sad, satisfaction with coping, nervous, worry, work, enjoyment, life, content, fatigued, feeling weak, listless, tired, trouble starting/finishing things, energy, able to do usual activities, sleep during the day, too tired to eat, frustrated being too tried to do usual activities, limited social activity due to tiredness, sleep during the day, headaches, short of breath, chest pain
FACT-F	Lack of energy, nausea, pain, treatment, feeling ill, time in bed, friends and family, sex life, sad, satisfaction with coping, nervous, worry, work, enjoyment, life, content, fatigued, feeling weak, listless, tired, trouble starting/finishing things, energy, able to do usual activities, sleep during the day, too tired to eat, frustrated being too tried to do usual activities, limited social activity due to tiredness
FSS^40^	Motivation low when fatigued, exercise brings on fatigue, easily fatigued, interferes with functioning and causes problems, interferes with work, disabling symptom
IPQ-R^41^	Illness, symptoms, consequences, emotional (sad, angry, worry, anxious, afraid, upset), psychological (stress/worry, negative thinking, family problems caused by illness, overwork, emotion state, lonely, anxious, empty), risk factors (hereditary, diet, poor medical care, behaviours), immunity, accident, pain, nausea, breathlessness, weight loss, fatigue, stiff joints, wheeziness, headaches, upset stomach, sleep difficulties, dizziness, loss of strength
KDQOL^42^	Daily activities (eg, housework, moving a table, pushing a vacuum cleaner, carrying groceries, climbing stairs, lifting heavy objects, bathing or dressing, bending, kneeling, or stooping), sport (eg, running, participating in strenuous sports, walking, bowling, or playing golf), social activities (eg, friends, family), work outside the home, sex life, travel, sleep
KDQOL-36^43^	Daily activities (eg, housework, moving a table, pushing a vacuum cleaner, carrying groceries, climbing stairs), bowling, or playing golf, social activities (eg, friends, family), work outside the home, sex life, travel
KDQOL-SF^44^	Daily activities (eg, housework, moving a table, pushing a vacuum cleaner, carrying groceries, climbing stairs, lifting heavy objects, bathing or dressing, bending, kneeling, or stooping), sport (eg, running, participating in strenuous sports, walking, bowling, or playing golf), social activities (eg, friends, family), work outside the home, sex life, travel, sleep
KSQ^20^	Itching, sleep disturbance/insomnia, loss of appetite, feeling tired, pain in bones/joints, poor concentration/mental alertness, loss of libido, loss of muscle strength/power, shortness of breath, cramp/muscle stiffness, restless legs, the need to urinate more often (night and/or day); feeling cold
LASA^45^	Social activities (friends, interaction, pleasure, relationships), physical well-being, fatigue
LUSS^20^	Loss of muscle strength/power, pain in joints/bones, muscle spasm/stiffness, excessive tiredness, sleep disturbance, poor concentration/mental alertness, restless legs, shortness of breath, impotence/lack of sex drive, loss of appetite, and itching
MOS^46^	Daily activities (eg, housework, moving a table, pushing a vacuum cleaner, carrying groceries, climbing stairs, lifting heavy objects, bathing or dressing, bending, kneeling, or stooping), travel, mobility limitations, assistance, ability to work (outside the home and housework), recreational/leisure activities, enjoyment, walking/movement, social activities (eg, friends, family), falling asleep, sleep not quiet, enough sleep, short of breath/headache, waken during sleep, snoring, naps, feeling drowsy or sleepy during the day
MOS-Sleep-R^47^	Falling asleep, sleep not quiet, enough sleep, short of breath/headache, waken during sleep, snoring, naps, feeling drowsy or sleepy during the day
PSQI^48^	Sleep (bed/wake time, hours), cough/snore, breathing, temperature, dreams, pain, medication, trouble staying awake, enthusiasm to get things done, bed partner/roommate
QIDS-SR^49^	Insomnia (sleep onset, mid-nocturnal, early morning), hypersomnia, mood, appetite (decreased, increased), weight (decreased, increased), concentration/decision making, outlook, suicidal ideation, involvement, energy/fatiguability, psychomotor (slowing, agitation)
SF-6D^50^	Daily activities (eg, housework, bathing, dressing), social activities, vigorous activities, work outside the home
SF-12^51^	Daily activities (eg, housework, moving a table, pushing a vacuum cleaner, climbing stairs, bowling, playing golf), social activities (eg, friends, family), work outside the home, downhearted, calm, energy levels
SF-36^52^	Daily activities (eg, housework, moving a table, pushing a vacuum cleaner, carrying groceries, climbing stairs, lifting heavy objects, bathing or dressing, bending, kneeling, or stooping), social activities (eg, friends, family), work outside the home, energy levels, tiredness, downhearted, worn out, nervous
Author developed	
Chen and Ku, 1998^53^	Fatigue
FS developed by Lin^54^	Decreased vigour and motivation, decreased physical ability, decreased mental ability, decreased daily activities, feeling down and lost control
Hao *et al* ^55^	Meeting family needs, enjoyment, ability to work (outside the home and housework), recreation/leisure, friends, family, relationships, sex life, lack of energy, sleep, time in bed, ill, nausea, side effects, quality of life, worry, coping, sad, support

BDI-I, The Beck Depression Inventory; CFQ, Chalder Fatigue Questionnaire; CKD-SI, Chronic Kidney Disease Symptom Index (Sri Lanka Version); 15D, 15 Dimensions; DSI, Dialysis Symptom Index; ESS, Epworth Sleepiness Scale; FACIT-Fatigue, Functional Assessment of Chronic Illness Therapy – Fatigue Scale; FACT-An, Functional Assessment of Cancer Therapy – Anemia; FACT-F, Functional Assessment of Chronic Illness Therapy – Fatigue; FSS, Fatigue Severity Scale; ICECAP-O, ICEpop CAPability measure for Older people; IPQ-R, Revised Illness Perception Questionnaire; KDQOL-36, Kidney Disease Quality of Life - 36-Item Questionnaire; KDQOL, Kidney Disease Quality of Life; KDQOL-SF, Kidney Disease Quality of Life - Short Form; KSQ, Kidney Symptom Questionnaire; LASA, Linear Analog Scale Assessment; LUSS, Leicester Uraemic Symptom Score; MOS, Medical Outcomes Study; MOS-Sleep-R, Medical Outcomes Study Sleep Scale Revised; PSQI, Pittsburgh Sleep Quality Index; QIDS-SR, Quick Inventory of Depressive Symptomatology-Self Report; SF-12, 12-Item Short Form Health Survey; SF-36, 36-Item Short Form Health Survey; SF-6D, Short-Form 6 Dimension.

### Psychometric properties

Of the 27 measures, only six have been validated in CKD not requiring KRT. The psychometric properties of these validated tools are provided in table 4 and online supplemental table S6. None of the tools were assessed across all psychometric domains and the validation data and psychometric properties that were evaluated varied.

**Table 4:** A summary of validation data of psychometric properties of measures that have been used to assess fatigue in CKD

Measure/psychometric properties	Content validity	Convergent validity	Known groups validity	Responsiveness	Test-retest reliability	Internal consistency	Total
CKD-SI		⚫️	⚫️		⚫️		3
FACT-An		⚫️	⚫️	⚫️	⚫️	⚫️	5
IPQ-R	⚫️					⚫️	2
KSQ	⚫️	⚫️					2
SF-6D		⚫️					1
SF-36		⚫️	⚫️	⚫️	⚫️	⚫️	5

Discriminant validity, structural validity, measurement error, criterion validity and cross-cultural validity were not reported in any of the validation studies of these measures.

CKD, chronic kidney disease; CKD-SI, Chronic Kidney Disease Symptom Index (Sri Lanka Version); FACT-An, Functional Assessment of Cancer Therapy – Anemia; IPQ-R, Revised Illness Perception Questionnaire; KSQ, Kidney Symptom Questionnaire; SF-36, 36-Item Short Form Health Survey; SF-6D, Short-Form 6 Dimension.

The CKD-SI symptom assessment measure, which is not specific to fatigue, is one of two tools designed specifically for CKD patients. It has demonstrated satisfactory convergent validity with each domain of the KDQOL.^17^ Known groups validity was evidenced by significantly higher CKD-SI scores in patients with CKD experiencing co-morbidities vs those without co-morbidities, and by negative correlation of symptom burden scores with estimated Glomerular Filtration Rate (eGFR).^17^ Test-retest reliability of the CKD-SI was high, with a Spearman’s r value of >0.9.^17^ The method of development (identifying items and reaching consensus) of this tool also supports content validity.

The Functional Assessment of Cancer Therapy – Anemia (FACT-An) tool was moderately correlated to the SF-36 vitality subscale.^18^ Internal consistency was high with Cronbach’s alpha ranging from 0.79 to 0.95. FACT-An demonstrated high test-retest reliability with an intraclass correlation coefficient ranging from 0.72 to 0.88^18^. Known groups validity was demonstrated with discrimination between groups (patients with varying levels of anaemia, using haemoglobin (Hb) levels at baseline) defined by SF-36 Physical Function and Vitality median split scores to FACT-An scores at baseline and FACT-An, FACT Anemia and FACT Fatigue subscale median split used to show a baseline difference to SF-36 scores.^18^

The Revised Illness Perception Questionnaire (IPQ-R) demonstrated unsatisfactory content validity with only 6 out of 31 patients agreeing with their total score; the remaining 25 disagreed with at least one subscale.^19^ The authors suggested the potential for some items to be modified for improved comprehension and relevance to people with CKD not requiring KRT. Internal consistency was moderate to high for all domains with Cronbach’s alpha ranging from 0.66 to 0.90^19^.

The KSQ demonstrated good content validity as no item received a poor relevance rating by patients.^20^ The mean of the Content Validity Index (CVI) scores of the whole questionnaire (0.81) fell within the recommended threshold^21^ (0.80).^20^ The frequency of 10 of the 13 items was negatively associated with the EQ-5D index score (total EQ-5D score=−0.648) indicating poor convergent validity (p value <0.002).^20^ However, convergent validity cannot be demonstrated solely by the relationship of symptom burden and health-related quality of life due to the small impact on total quality of life.

The Short form – 6 Dimension (SF-6D) vitality domain was weakly correlated with the ICEpop CAPability measure for Older people (ICECAP-O) role and control domains (0.41, 0.42, p<0.001 respectively).^22^

The SF-36 vitality domain demonstrated strong convergent validity with the FACT-Fatigue and Anemia subscales (r=0.76, r=0.77 respectively).^23^ Additionally, responsiveness was seen in the vitality domain in both the dialysis and non-dialysis group with improvements seen by weeks 9 or 17 compared with baseline.^23^

## Discussion

Fatigue has been identified by patients, caregivers and health professionals as an outcome of critical importance to patients with CKD not requiring KRT.^9^ However, it is reported infrequently in trials and observational studies in patients with CKD not requiring kidney replacement therapy. Our results indicate that fatigue has been assessed using 27 different tools across the 97 studies identified, with more than half of the tools (63%) used in only one study. Most of the tools (21 (78%)) had not been validated to assess fatigue in the specific patient population of interest: those with CKD not requiring KRT. For the limited number of tools that had been validated in this population, the evidence to support psychometric robustness (reliability and validity) relevant to patients with CKD was either incomplete or not reported. Convergent validity was the most commonly assessed property, followed by internal consistency. Structural validity, criterion validity, cross-cultural validity and measurement error were not assessed by any of the tools.

The SF-36 and KDQOL-SF were the most frequently used tools for fatigue in patients with CKD not requiring KRT. However, the KDQOL-SF was not developed for early-stage CKD prior not requiring KRT, and the SF-36 has only limited validation data. Both these tools included only a limited number of content dimensions and only frequency of fatigue under measurement dimensions. The recently developed Kidney Symptom Questionnaire includes the top 13 symptoms chosen by patients with CKD not requiring kidney replacement therapy including fatigue.^20^ No tool fulfils all the requirements to be considered as a patient-reported core outcome measure for fatigue as they are either too long (limiting feasibility), unvalidated (psychometric properties have not been established for CKD) thus limiting their utility as a PROM. However, the tools do provide important insights and potential tools to be put forward when designing and/or validating a new measure.

The tools varied in length, complexity and content. The number of items ranged from 3 to 134, and completion time from less than 2 minutes to 30 minutes. 15 (56%) tools were available in a language other than English (including Arabic, Chinese, Danish, Dutch, German, Spanish, Japanese). Translations and cultural adaptations are key to establish an appropriate and valid tool for transferability into non-English speaking populations, enhancing the depth and understanding of fatigue in CKD patients. This helps increase generalisability, reduce missing data and sample attrition.^24^ Over half (15, 56%) of the tools assessed the severity of fatigue and 13 (48%) assessed the frequency of fatigue. Only two tools assessed both the severity and frequency of fatigue. The most common content dimensions were tiredness, level of energy and muscle weakness. The meaning of and impact of fatigue on CKD patients has not been assessed and remains uncertain. However, a systematic review and thematic analysis of qualitative studies on patient perspectives on the meaning and impact of fatigue in haemodialysis, identified four key experiences including the debilitating and exhausting burden of dialysis, restricted life participation, diminishing capacities to fulfil relationship roles and vulnerable to misunderstanding.^13^

Fatigue has been identified as a critically important outcome for trials in patients receiving haemodialysis^25^ and an important outcome for trials in CKD,^2^ kidney transplant recipients,^26^ patients receiving peritoneal dialysis,^27^ and patients with polycystic kidney disease^28^ and glomerular disease.^29 30^ Despite this, fatigue is infrequently and inconsistently reported. Similarly to our findings, in a systematic review of tools for fatigue used in research in patients receiving haemodialysis, 45 different tools were identified, with SF-36 (22, 16%) and KDQOL-SF (17, 12%) being the most frequently used.^10^ We found that five tools for fatigue (CFQ, FACIT-Fatigue, FSS, KDQOL-SF, SF-36) have been used in studies in both patients with CKD and patients receiving haemodialysis. Inconsistent reporting limits the comparability across studies and diagnoses. Psychometrically robust tools in each treatment stage of kidney disease including CKD not requiring KRT will provide confidence in the validity and reliability of results as it cannot be assumed that tools will be suitable across different populations.

This review comprehensively identified PROMs relevant to fatigue in patients with CKD that have been reported in trials and observational studies. However, there are some potential limitations. As our explicit focus was to assess the characteristics and psychometric properties rather than the effect of interventions, we did not conduct a risk of bias assessment. We acknowledge that there may be other tools of fatigue that have not been included in our review, that could be potentially appropriate for patients with CKD. We did not review documents and studies related to the primary development of the instrument, only the reporting of psychometric properties relating to CKD. The CKD focused search strategy would not have identified the full extent of cultural adaptations and translations available for the tool’s availability.

There is a need for a standardised, validated and reliable PROM for fatigue for patients with CKD not requiring KRT to ensure this outcome of importance to patients, caregivers and health professionals can be consistently, accurately and meaningfully assessed. Systematically measuring fatigue in patients with CKD across using a standardised measure will enable assessment of the comparative effect of interventions. Additionally, trials that incorporate PROMs increase the impact in policy and practice through improving its relevance, reliability and value.^31^ A feasible measure should be short, yet broadly capture individual circumstances.

The international SONG-CKD initiative identified fatigue as an important outcome for patients, caregivers and health professionals.^2^ Of note, the SONG-HD Fatigue measure has been developed and validated for use in patients receiving haemodialysis.^32^ The SONG-HD Fatigue measure assesses fatigue across the past week on a 4-point Likert scale with three items: (1) did you feel tired? (2) did you lack energy? and (3) did fatigue limit your usual activities? Further work is required to identify and validate a fatigue measure that addresses dimensions important to patients with CKD not requiring KRT with due consideration of the SONG-HD Fatigue measure. Validating a PROM for fatigue in patients with CKD not requiring KRT will involve an international multi-stakeholder consensus workshop, along with pilot and validation studies. Additionally, further work will also be undertaken to ensure language and cross-cultural validity.

The evidence regarding fatigue in patients with CKD not yet requiring KRT is lacking, and our findings highlight the need to include PROMs for fatigue in trials and observational studies. Implementing content-relevant and validated PROMs in research provides stronger evidence to better support shared decision-making and ultimately improve efforts to manage fatigue in patients with CKD.

## Supplementary material

10.1136/bmjopen-2025-099592 online supplemental file 1

## Data Availability

All data relevant to the study are included in the article or uploaded as supplementary information.
