# Supplementary material for: Patient-reported outcome measures for fatigue in patients with chronic kidney disease: a systematic review
Source: BMJ Open. 2025 Jul 16;15(7):e099592. doi: 10.1136/bmjopen-2025-099592 (PMC12273140; doi:10.1136/bmjopen-2025-099592)
Supplement: online supplemental file 1 [file bmjopen-15-7-s001.docx]

**SUPPLEMENTARY MATERIAL**

Table S1. PRISMA Checklist

Table S2. Search strategies

Table S3. Overview of all measurement properties

Table S4. Characteristics of randomized, non-randomized and observational studies

Table S5. Validation data of psychometric properties of measures that have been used to assess fatigue in CKD

**Table S1. PRISMA Checklist**

| **Section and Topic** | **Item #** | **Checklist item** | **Location where item is reported (P#)** |
| --- | --- | --- | --- |
| **TITLE** | | | |
| Title | 1 | Identify the report as a systematic review. | 1 |
| **ABSTRACT** | | | |
| Abstract | 2 | See the PRISMA 2020 for Abstracts checklist. | 4 |
| **INTRODUCTION** | | | |
| Rationale | 3 | Describe the rationale for the review in the context of existing knowledge. | 6 |
| Objectives | 4 | Provide an explicit statement of the objective(s) or question(s) the review addresses. | 6 |
| **METHODS** | | | |
| Eligibility criteria | 5 | Specify the inclusion and exclusion criteria for the review and how studies were grouped for the syntheses. | 7 |
| Information sources | 6 | Specify all databases, registers, websites, organisations, reference lists and other sources searched or consulted to identify studies. Specify the date when each source was last searched or consulted. | 7 |
| Search strategy | 7 | Present the full search strategies for all databases, registers and websites, including any filters and limits used. | 7, Supplement |
| Selection process | 8 | Specify the methods used to decide whether a study met the inclusion criteria of the review, including how many reviewers screened each record and each report retrieved, whether they worked independently, and if applicable, details of automation tools used in the process. | 7 |
| Data collection process | 9 | Specify the methods used to collect data from reports, including how many reviewers collected data from each report, whether they worked independently, any processes for obtaining or confirming data from study investigators, and if applicable, details of automation tools used in the process. | 7, 8 |
| Data items | 10a | List and define all outcomes for which data were sought. Specify whether all results that were compatible with each outcome domain in each study were sought (e.g. for all measures, time points, analyses), and if not, the methods used to decide which results to collect. | 8 |
|  | 10b | List and define all other variables for which data were sought (e.g. participant and intervention characteristics, funding sources). Describe any assumptions made about any missing or unclear information. | 8 |
| Study risk of bias assessment | 11 | Specify the methods used to assess risk of bias in the included studies, including details of the tool(s) used, how many reviewers assessed each study and whether they worked independently, and if applicable, details of automation tools used in the process. | N/A |
| Effect measures | 12 | Specify for each outcome the effect measure(s) (e.g. risk ratio, mean difference) used in the synthesis or presentation of results. | N/A |
| Synthesis methods | 13a | Describe the processes used to decide which studies were eligible for each synthesis (e.g. tabulating the study intervention characteristics and comparing against the planned groups for each synthesis (item #5)). | N/A |
|  | 13b | Describe any methods required to prepare the data for presentation or synthesis, such as handling of missing summary statistics, or data conversions. | N/A |
|  | 13c | Describe any methods used to tabulate or visually display results of individual studies and syntheses. | N/A |
|  | 13d | Describe any methods used to synthesize results and provide a rationale for the choice(s). If meta-analysis was performed, describe the model(s), method(s) to identify the presence and extent of statistical heterogeneity, and software package(s) used. | 7, 8 |
|  | 13e | Describe any methods used to explore possible causes of heterogeneity among study results (e.g. subgroup analysis, meta-regression). | N/A |
|  | 13f | Describe any sensitivity analyses conducted to assess robustness of the synthesized results. | N/A |
| Reporting bias assessment | 14 | Describe any methods used to assess risk of bias due to missing results in a synthesis (arising from reporting biases). | N/A |
| Certainty assessment | 15 | Describe any methods used to assess certainty (or confidence) in the body of evidence for an outcome. | N/A |
| **RESULTS** | | | |
| Study selection | 16a | Describe the results of the search and selection process, from the number of records identified in the search to the number of studies included in the review, ideally using a flow diagram. | 9, 23 |
|  | 16b | Cite studies that might appear to meet the inclusion criteria, but which were excluded, and explain why they were excluded. | N/A |
| Study characteristics | 17 | Cite each included study and present its characteristics. | 24-26 |
| Risk of bias in studies | 18 | Present assessments of risk of bias for each included study. | N/A |
| Results of individual studies | 19 | For all outcomes, present, for each study: (a) summary statistics for each group (where appropriate) and (b) an effect estimate and its precision (e.g. confidence/credible interval), ideally using structured tables or plots. | N/A |
| Results of syntheses | 20a | For each synthesis, briefly summarise the characteristics and risk of bias among contributing studies. | N/A |
|  | 20b | Present results of all statistical syntheses conducted. If meta-analysis was done, present for each the summary estimate and its precision (e.g. confidence/credible interval) and measures of statistical heterogeneity. If comparing groups, describe the direction of the effect. | N/A |
|  | 20c | Present results of all investigations of possible causes of heterogeneity among study results. | N/A |
|  | 20d | Present results of all sensitivity analyses conducted to assess the robustness of the synthesized results. | N/A |
| Reporting biases | 21 | Present assessments of risk of bias due to missing results (arising from reporting biases) for each synthesis assessed. | N/A |
| Certainty of evidence | 22 | Present assessments of certainty (or confidence) in the body of evidence for each outcome assessed. | N/A |
| **DISCUSSION** | | | |
| Discussion | 23a | Provide a general interpretation of the results in the context of other evidence. | 14-15 |
|  | 23b | Discuss any limitations of the evidence included in the review. | 15 |
|  | 23c | Discuss any limitations of the review processes used. | 15 |
|  | 23d | Discuss implications of the results for practice, policy, and future research. | 15-16 |
| **OTHER INFORMATION** | | | |
| Registration and protocol | 24a | Provide registration information for the review, including register name and registration number, or state that the review was not registered. | N/A* |
|  | 24b | Indicate where the review protocol can be accessed, or state that a protocol was not prepared. | N/A* |
|  | 24c | Describe and explain any amendments to information provided at registration or in the protocol. | N/A* |
| Support | 25 | Describe sources of financial or non-financial support for the review, and the role of the funders or sponsors in the review. | 2 |
| Competing interests | 26 | Declare any competing interests of review authors. | 2 |
| Availability of data, code and other materials | 27 | Report which of the following are publicly available and where they can be found: template data collection forms; data extracted from included studies; data used for all analyses; analytic code; any other materials used in the review. | 3, Supplement |

*Not registered as Prospero did not accept reviews of patient-reported outcome measures at the time of search

**Table S2. Search strategies**

**Embase 1974 to February 07, 2023**

| Searches |
| --- |
| 1. exp chronic kidney failure/ |
| 1. chronic kidney disease.tw. |
| 1. pre-dialysis.tw. |
| 1. or/1-3 |
| 1. exp clinical trial/ or exp controlled study/ or exp randomized controlled trial/ |
| 1. trial.tw. |
| 1. exp epidemiology/ |
| 1. case control.tw. |
| 1. cohort study.tw. |
| 1. exp observational study/ |
| 1. exp prospective study/ |
| 1. exp cross-sectional study/ |
| 1. or/5-12 |
| 1. exp “quality of life”/ |
| 1. QOL.tw. |
| 1. exp Sickness Impact Profile/ |
| 1. exp daily life activity/ |
| 1. activities of life.tw. |
| 1. activities of liv$.tw. |
| 1. exp leisure/ |
| 1. exp travel/ |
| 1. exp work capacity/ or exp work/ or exp return to work/ |
| 1. exp school/ |
| 1. exp employment/ |
| 1. life participation.tw. |
| 1. life involvement.tw. |
| 1. daily activit$.tw. |
| 1. exp fatigue/ |
| 1. fatigue.tw. |
| 1. exp chronic fatigue syndrome/ |
| 1. (weary or weariness or exhaust$).tw. |
| 1. tired$.tw. |
| 1. lethargy$.tw. |
| 1. (energy$ or vigor$ or vigour$).tw. |
| 1. exp Sleep/ |
| 1. sleep.tw. |
| 1. or/14-36 |
| 1. 4 and 13 and 37 |
| 1. exp short survey/ or exp health survey/ |
| 1. exp structured questionarie/ or exp questionnaire/ |
| 1. survey$.tw. |
| 1. questionnaire$.tw. |
| 1. exp patient-reported outcome/ |
| 1. patient-reported outcome$.tw. |
| 1. PROM$.tw. |
| 1. index$.tw. |
| 1. instrument.tw. |
| 1. or/39-47 |
| 1. 38 and 48 |

**MEDLINE 1946 to February 07, 2023**

| Searches |
| --- |
| 1. exp chronic kidney disease/ |
| 1. chronic kidney disease.tw. |
| 1. exp Renal Insufficiency, Chronic/ or exp Kidney Failure, Chronic/ |
| 1. pre-dialysis.tw. |
| 1. or/1-4 |
| 1. Randomized controlled trial.pt. |
| 1. controlled clinical trial.pt. |
| 1. exp Clinical Trial/ |
| 1. exp epidemiologic studies/ |
| 1. case control.tw. |
| 1. (cohort adj stud*).tw. |
| 1. exp Observational Study/ |
| 1. exp Prospective Studies/ |
| 1. exp Cross-Sectional Studies/ |
| 1. or/6-14 |
| 1. exp quality of life/ |
| 1. QOL.tw. |
| 1. exp sickness impact profile/ |
| 1. exp activities of daily living/ |
| 1. exp leisure activities/ |
| 1. exp travel/ |
| 1. exp work/ |
| 1. exp school/ |
| 1. activities of daily living$.tw. |
| 1. exp employment/ |
| 1. life participation.tw. |
| 1. life involvement.tw. |
| 1. daily activitie$.tw. |
| 1. exp fatigue/ |
| 1. fatigue.tw. |
| 1. chronic fatigue syndrome.tw. |
| 1. (weary or weariness or exhaust$).tw. |
| 1. tired$.tw. |
| 1. letharg$.tw. |
| 1. (energy$ or vigor$ or vigour$).tw. |
| 1. exp Sleep/ |
| 1. sleep.tw. |
| 1. exp Muscle Fatigue/ or exp Mental Fatigue/ |
| 1. or/16-38 |
| 1. 5 and 15 and 39 |
| 1. exp "Surveys and Questionnaires"/ |
| 1. (survey$ or questionnaire$).tw. |
| 1. exp Patient Outcome Assessment/ |
| 1. exp Patient Reported Outcome Measures/ |
| 1. PROM$.tw. |
| 1. patient-reported$.tw. |
| 1. exp Health Surveys/ |
| 1. (index$ or score$).tw. |
| 1. instrument$.tw. |
| 1. or/41-49 |
| 1. 40 and 50 |

**PsycINFO 1967 to 7^th^ February 2023**

| Searches |
| --- |
| 1. exp Kidney Diseases/ |
| 1. chronic kidney disease.tw. |
| 1. chronic kidney failure.tw. |
| 1. or/1-3 |
| 1. exp "Quality of Life"/ |
| 1. exp "Activities of Daily Living"/ |
| 1. daily living$.tw. |
| 1. exp Leisure Time/ or exp Recreation/ |
| 1. exp Traveling/ |
| 1. exp Work-Life Balance/ |
| 1. exp Schools/ |
| 1. study$.tw. |
| 1. exp Employment Status/ |
| 1. exp Participation/ |
| 1. life participat$.tw. |
| 1. life involvement$.tw. |
| 1. exp Chronic Fatigue Syndrome/ or exp Fatigue/ |
| 1. fatigue.tw. |
| 1. (weary or weariness or exhaust$).tw. |
| 1. tired$.tw. |
| 1. letharg$.tw. |
| 1. (energy$ or vigor$ or vigour$).tw. |
| 1. exp Sleep Deprivation/ or exp Sleep/ |
| 1. sleep$.tw. |
| 1. or/5-24 |
| 1. 4 and 25 |
| 1. exp Mail Surveys/ or exp Telephone Surveys/ or exp Surveys/ or exp Online Surveys/ |
| 1. survey$.tw. |
| 1. exp Questionnaires/ |
| 1. questionnaire$.tw. |
| 1. (index$ or score$).tw. |
| 1. instrument$.tw. |
| 1. exp Patient Reported Outcome Measures/ |
| 1. patient-reported$.tw. |
| 1. or/27-34 |
| 1. 26 and 35 |

**CINAHL**

| Search ID | Search terms |
| --- | --- |
| S1 | (MH "Kidney Failure, Chronic+") OR (MH "Renal Insufficiency, Chronic+") |
| S2 | (MH "Fatigue+") OR (MH "Fatigue Syndrome, Chronic") OR (MH "Mental Fatigue+") |
| S3 | (MH "Activities of Daily Living+") |
| S4 | S2 OR S3 |
| S5 | S1 AND S4 |

**Table S3. Overview of all measurement properties**

| Measurement | Definition according to the COSMIN* taxonomy |
| --- | --- |
| Hypotheses testing | The extent of scores being consistent with the hypotheses ensuring there is measurement and construct validity. |
| Responsiveness | The ability of the measure to detect changes over time. |
| Internal consistency | The degree of correlation between different items in the measure. |
| Measurement error | Errors of scores, either systematic or random, that are not the result of true changes in the construct being measured. |
| Reliability | The reproducibility and consistency of a measure that is free from measurement error. |
| Construct validity | The extent to which the measure assesses the intended outcome. |
| Convergent validity | The extent to which two theoretically related measures have correlated results. |
| Discriminant validity | The extent to which two theoretically unrelated measures have dissimilar results. |
| Content validity (including face validity) | The extent to which the measure adequately reflects the construct being assessed. |
| Structural validity | The extent to which the instruments measurement scores reflect the dimension of the construct being measured. |
| Criterion validity | The extent to which the measure correlates to a gold standard. |
| Cross-cultural validity | The extent to which the performance of the outcomes of a translated or culturally adapted instrument adequately reflects the performance of the original version of the instrument. |

^*^ COnsensus-based Standards for the selection of health Measurement INstruments

**Table S4. Characteristics of randomized, non-randomized and observational studies**

**Table S4 a. Characteristics of randomized controlled trials**

| Study | N | Country | Intervention | Measure used |
| --- | --- | --- | --- | --- |
| Akizawa 2011 ^1^ | 322 | Japan | A high Hb group to receive darbepoetin alfa (DA) or a low Hb group to receive recombinant human erythropoietin (rHuEPO). | SF-36 |
| Alexander 2007 ^2^ | 81 | US | Receiving darbepoetin alfa in addition to conversative management for CKD. | SF-36, KDQOL, Katz ADL and Lawton IADL |
| Blakeman 2014 ^3^ | 436 | UK | Kidney information guidebook: a booklet and interactive website that tailored access to community resources; and telephone-guided help from a lay health worker. | MOS |
| Bohlke 2022 ^4^ | 150 | Brazil | 16 week physical training | SF-36 |
| Campbell 2008 ^5^ | 53 | Australia | Nutrition | KDQOL-SF |
| Greenwood 2022 ^6^ | 74 | UK | Intravenous iron therapy | WSAS |
| Hedayati 2017 ^7^ | 201 | US | Treatment with sertraline to improve depressive symptoms | WSAS, KDQOL-SF |
| Hirakata 2010 ^8^ | 171 | Japan | A higher Hb target (12–13 g/dl) by darbepoetin alfa (DPO) was compared with the conventional Hb target by epoetin alfa (EPO). | SF-36 |
| Lewis 2011 ^9^ | 4038 | US, Canada | Darbepoetin Alfa | SF-36 |
| Li 2020 ^10^ | 49 | Taiwan | Effectiveness of wearable devices, a health management platform, and social media. | KDQOL-SF |
| Magar 2017 ^11^ | 120 | Canada | Daily compared to monthly vitamin D supplementation | SF-36 |
| Mustata 2011 ^12^ | 20 | Canada | Exercise | SF-36 |
| Provenzano 2005 ^13^ | 519 | US | 10,000 units (U) once weekly (QW), 20,000 U every two weeks (Q2W), 30,000 U every three weeks (Q3W) or 40,000 U every four weeks (Q4W). | LASA |
| Roger 2014 ^14^ | 51 | Australia, Canada | Darbepoetin alfa | SF-36, FACT-An |
| Rossi 2014 ^15^ | 96 | US | Guided exercise two times per week for 12 weeks. | RAND-36 |
| Shin 2022 ^16^ | 149 | Republic of Korea/South Korea | AST-120 | KDQOL-SF |
| Uchiyama 2021 ^17^ | 46 | Japan | Exercise group patients performed aerobic exercise at 40–60% peak heart rate thrice weekly and resistance training at 70% of one-repetition maximum twice weekly at home for 6 months. | KDQOL-SF |
| van Craenenbroeck 2015 ^18^ | 40 | Belgium | 3-month home-based aerobic training program consisted of 4 daily cycling sessions of 10 minutes each at a target heart rate, calculated as 90% of the heart rate achieved at the anaerobic threshold. | KDQOL-SF |
| Villar 2011 ^19^ | 89 | France | Group 1 patients were assigned to a subnormal Hb level range (110–129 g/l) and group 2 to a normal Hb level range (130–149 g/l). | SF-36 |
| Yu-Hsiu 2012 ^20^ | 94 | Taiwan | Exercise education intervention | Author developed (Chen and Ku, 1998) |

**Table S4 b. Characteristics of non-randomized controlled trials**

| Study | N | Country | Intervention | Measure used |
| --- | --- | --- | --- | --- |
| Guo 2022 ^21^ | 49 | China | Non-protein energy supplement | SF-36 |
| Lefebvre 2006 ^22^ | 1326 | US, Canada | Once-weekly (QW) dosing of epoetin alfa for the treatment of anemia in non-dialysis CKD patients | LASA, KDQ |

**Table S4 c. Characteristics of observational studies**

| Study | N | Country | Measure used |
| --- | --- | --- | --- |
| Abeywickrama 2020 ^23^ | 120 | Sir Lanka | KDQOL-SF |
| Adame Perez 2019 ^24^ | 41 | Canada | SF-36 |
| Aggarwal 2016 ^25^ | 200 | India | SF-36 |
| Arnold 2022 ^26^ | 109 | Australia | SF-36 |
| Bagasha 2021 ^27^ | 364 | Uganda | KDQOL-SF |
| Bonner 2013 ^28^ | 28 | Australia | SF-36, HAP |
| Brown 2017 ^29^ | 283 | UK | LUSS |
| Canales 2020 ^30^ | 248 | US | ESS |
| Chan 2016 ^31^ | 141 | China | ESS |
| Chesnaye 2022 ^32^ | 1421 | Germany, Italy, The Netherlands, Poland, Sweden, and the UK. | SF-36 |
| Chin 2008 ^33^ | 944 | Korea | SF-36 |
| Davison 2009 ^34^ | 185 | Canada | SF-6D |
| de Goeij 2014 ^35^ | 436 | The Netherlands | IPQ-R |
| Delgado 2009 ^36^ | 293 | Colombia | SF-36 |
| Eltawdy 2016 ^37^ | 40 | Egypt | ESS |
| Erez 2016 ^38^ | 74 | England | SF-36 |
| Farag 2011 ^39^ | 69 | US | KDQOL-SF |
| Faye 2022 ^40^ | 2787 | France | KDQOL-36 |
| Feng 2013 ^41^ | 362 | Singapore | SF-12 |
| Finkelstein 2009 ^42^ | 1186 | North America | KDQOL-SF |
| Finkelstein 2018 ^43^ | 204 | US | SF-36 |
| Gregg 2019 ^44^ | 266 | US | QIDS-SR, SF-12, BDI-I |
| Guedes 2021 ^45^ | 2513 | Brazil, France, US | KDQOL-36 |
| Gunawardena 2020 ^46^ | 1079 | Sir Lanka | KDQOL-SF |
| Gyamlani 2011 ^47^ | 71 | US | SF-36 |
| Hakamaki 2021 ^48^ | 210 | Finland | KDQOL-SF |
| Hamilton 2006 ^49^ | 34 | US | SF-36 |
| Hanson 2009 ^50^ | 79 | US | KDQOL-SF |
| Hao 2021 ^51^ | 456 | China | Hao et al |
| Ho 2022 ^52^ | 242 | Taiwan | FS was developed by Lin et al. |
| Hoshino 2020 ^53^ | 2465 | Brazil, France, Germany, Japan, and the US | KDQOL-36 |
| Iliescu 2004 ^54^ | 120 | Canada | PSQI |
| Jalal 2022 ^55^ | 180 | Saudi Arabia | KDQOL-SF |
| Jhamb 2014 ^56^ | 173 | US | SF-36 |
| Kaltsouda 2011 ^57^ | 98 | Greece | SF-36 |
| Kefale 2019 ^58^ | 256 | Ethiopia | SF-36 |
| Kharshid 2020 ^59^ | 526 | Malaysia | SF-36 (RAND-36) |
| Kim 2023 ^60^ | 970 | Korea | KDQOL-SF |
| Khuston 2018 ^61^ | 432 | US | ESS |
| Korevaar 2000 ^62^ | 301 | The Netherlands | SF-36, EQ-5D-3L (EuroQol-3) |
| Krishnasamy 2016 ^63^ | 108 | Australia | SF-12 |
| Kularatna 2019 ^64^ | 1096 | Sir Lanka | EQ-5D-3L, SF-6D |
| Kumar 2010 ^65^ | 830 | US | PSQI, KDQOL |
| Kusek 2002 ^66^ | 1094 | US | SF-36 |
| Lemos 2015 ^67^ | 170 | Brazil | SF-36 |
| Lin 2022 ^68^ | 497 | Taiwan | SF-12 |
| MacDonald 2012 ^69^ | 26 | UK | SF-36, FACIT-fatigue |
| Mansur 2014 ^70^ | 61 | Brazil | SF-36 |
| Martini 2018 ^71^ | 140 | Brazil | SF-36, Author developed measure for Scale of BADL |
| McKercher 2013 ^72^ | 49 | Australia | KDQOL-SF, EQ-5D |
| Mujais 2009 ^73^ | 1186 | US | KDQOL-SF |
| Nicholl 2012 ^74^ | 119 | Canada | ESS |
| Nixon 2020 ^75^ | 353 | UK | KSQ |
| Oh 2019 ^76^ | 1622 | Korea | KDQOL-SF |
| Oh 2022 ^77^ | 1618 | Korea | KDQOL-SF |
| Oh 2017 ^78^ | 1844 | Korea | KDQOL-SF |
| Okoro 2022 ^79^ | 220 | Nigeria | 15D |
| Peng 2013 ^80^ | 57 | China | SF-36 |
| Pereira 2022 ^81^ | 33 | Brazil | SF-36 |
| Perlman 2005 ^82^ | 634 | US | SF-36 |
| Picard 2021 ^83^ | 50 | Canada | SF-36 |
| Rajan 2013 ^84^ | 22,273 | US | SF-36 |
| Ruszkowski 2021 ^85^ | 100 | Poland | MOS-Sleep-R |
| Seidel 2014 ^86^ | 173 | Germany | SF-36, LLFDI |
| Senanayake 2020 ^87^ | 1079 | Sir Lanka | KDQOL-SF |
| Tanner 2019 ^88^ | 202 | Ghana | RAND-36 |
| Taptagaporn 2021 ^89^ | 258 | Thailand | KDQOL-SF |
| Tesfaye 2020 ^90^ | 101 | Australia | Katz ADL, Lawton and Brody IADL, KDQOL-36 |
| Wan Zukiman 2017 ^91^ | 187 | Malaysia | DSI |
| Wang 2019 ^92^ | 1079 | China | KDQOL-36 |
| Wee 2016 ^93^ | 311 | Singapore | KDQOL-SF |
| Wirkner 2022 ^94^ | 160 | Germany | KDQOL-SF |
| Wulczyn 2022 ^95^ | 3685 | US | KDQOL-36 |
| Xiong 2022 ^96^ | 558 | China | SF-36 |
| Yapa 2021 ^97^ | 886 | Sir Lanka | SF-36 |

**Table S5. Dimension of fatigue assessed by each measure: A visual.**

| Measure | Measurement Dimensions | | | | Content Dimensions | | | | | | | | | | Total |
| --- | --- | --- | --- | --- | --- | --- | --- | --- | --- | --- | --- | --- | --- | --- | --- |
|  | Severity | Frequency | Duration | Change | Tiredness | Muscle Weakness | Level of Energy | Ability to Think Clearly | Ability to Concentrate | Verbal Abilities | Motivation | Memory | Negative Emotions | Life Participation |  |
| 15D (33) | **⚫️** |  |  |  |  |  |  | **⚫️** |  | **⚫️** |  | **⚫️** | **⚫️** |  | 5 |
| BDI-I (34) | **⚫️** |  |  | **⚫️** | **⚫️** |  | **⚫️** |  | **⚫️** |  | **⚫️** |  | **⚫️** |  | 7 |
| CFQ (35) | **⚫️** |  |  |  | **⚫️** | **⚫️** | **⚫️** |  | **⚫️** | **⚫️** | **⚫️** | **⚫️** |  |  | 7 |
| CKD-SI (17) | **⚫️** |  |  |  | **⚫️** | **⚫️** | **⚫️** |  |  |  | **⚫️** | **⚫️** |  |  | 6 |
| DSI (36) | **⚫️** |  |  |  | **⚫️** |  | **⚫️** |  |  |  |  |  |  |  | 3 |
| ESS (37) | **⚫️** |  |  |  | **⚫️** |  |  |  | **⚫️** |  |  |  |  |  | 3 |
| FACIT-Fatigue (38) | **⚫️** |  |  |  | **⚫️** | **⚫️** | **⚫️** |  |  |  | **⚫️** |  | **⚫️** | **⚫️** | 7 |
| FACT-An (39) | **⚫️** |  |  |  | **⚫️** | **⚫️** | **⚫️** |  |  |  | **⚫️** |  | **⚫️** | **⚫️** | 7 |
| FACT-F | **⚫️** |  |  |  | **⚫️** | **⚫️** | **⚫️** |  |  |  | **⚫️** |  | **⚫️** | **⚫️** | 7 |
| FSS (40) | **⚫️** |  |  |  |  |  |  |  |  |  | **⚫️** |  |  | **⚫️** | 3 |
| IPQ-R (41) | **⚫️** |  |  |  | **⚫️** | **⚫️** |  |  |  |  |  |  | **⚫️** |  | 4 |
| KDQOL (42) |  | **⚫️** |  |  | **⚫️** | **⚫️** | **⚫️** |  |  |  |  |  |  |  | 4 |
| KDQOL-36 (43) |  | **⚫️** |  |  | **⚫️** | **⚫️** | **⚫️** |  |  |  |  |  |  |  | 4 |
| KDQOL-SF (44) |  | **⚫️** |  |  | **⚫️** | **⚫️** | **⚫️** |  |  |  |  |  |  |  | 4 |
| KSQ (20) |  | **⚫️** |  |  | **⚫️** | **⚫️** |  |  | **⚫️** |  |  |  |  |  | 4 |
| LASA (45) | **⚫️** |  |  |  | **⚫️** |  |  |  |  |  |  |  |  |  | 2 |
| LUSS (20) |  | **⚫️** |  |  | **⚫️** | **⚫️** |  |  | **⚫️** |  |  |  |  |  | 4 |
| MOS (46) |  | **⚫️** |  |  | **⚫️** |  | **⚫️** |  |  |  |  |  |  | **⚫️** | 4 |
| MOS-Sleep-R (47) |  | **⚫️** |  |  | **⚫️** |  |  |  |  |  |  |  |  |  | 2 |
| PSQI (48) | **⚫️** | **⚫️** |  |  | **⚫️** |  |  |  |  |  | **⚫️** |  |  |  | 4 |
| QIDS-SR (49) | **⚫️** |  |  |  | **⚫️** |  | **⚫️** |  | **⚫️** |  |  |  |  |  | 4 |
| SF-6D (50) |  | **⚫️** |  |  |  |  | **⚫️** |  |  |  |  |  |  |  | 2 |
| SF-12 (51) |  | **⚫️** |  |  |  |  | **⚫️** |  |  |  |  |  |  |  | 2 |
| SF-36 (52) |  | **⚫️** |  |  | **⚫️** |  | **⚫️** |  |  |  |  |  |  |  | 3 |
| Author developed | | | | | | | | | | | | | | | |
| Chen and Ku, 1998 (53) |  | **⚫️** |  |  | **⚫️** |  |  |  |  |  |  |  |  |  | 2 |
| FS developed by Lin (54) |  | **⚫️** |  |  |  |  |  | **⚫️** | **⚫️** |  | **⚫️** |  | **⚫️** | **⚫️** | 6 |
| Hao et al (55) | **⚫️** |  |  |  |  |  | **⚫️** |  |  |  | **⚫️** |  | **⚫️** |  | 4 |

15D: 15 Dimensions; BDI-I: The Beck Depression Inventory; CFQ: Chalder Fatigue Questionnaire; CKD-SI: Chronic Kidney Disease Symptom Index) Sri Lanka Version; DSI: Dialysis Symptom Index; ESS: Epworth Sleepiness Scale; FACIT-Fatigue: Functional Assessment of Chronic Illness Therapy – Fatigue Scale; FACT-An: Functional Assessment of Cancer Therapy – Anemia; FACT-F: Functional Assessment of Chronic Illness Therapy – Fatigue; FSS: Fatigue Severity Scale; ICECAP-O: ICEpop CAPability measure for Older people; IPQ-R: Revised Illness Perception Questionnaire; KDQOL: Kidney Disease Quality of Life; KDQOL-36: Kidney Disease Quality of Life - 36-Item Questionnaire; KDQOL-SF: Kidney Disease Quality of Life - Short Form; KSQ: Kidney Symptom Questionnaire; LASA: Linear Analog Scale Assessment; LUSS: Leicester Uraemic Symptom Score; MOS: Medical Outcomes Study; MOS-Sleep-R: Medical Outcomes Study Sleep Scale Revised; PSQI: Pittsburgh Sleep Quality Index; QIDS-SR: Quick Inventory of Depressive Symptomatology-Self Report; SF-6D: Short-Form 6 Dimension; SF-12: 12-Item Short Form Health Survey; SF-36: 36-Item Short Form Health Survey

**Table S6. Validation data of psychometric properties of measures that have been used to assess fatigue in CKD**

| Measure | Validity | Reliability |
| --- | --- | --- |
| CKD-SI ^98^ | **Known groups validity:** Symptom burden was assessed in 250 CKD patients with comorbid (37.0; IQR 23.0-52.0) and no comorbid conditions (29.5; IQR 16.0-46.0) with the Mann-Whitney U Test (p<0.001). It was also assessed in patients aged below or equal to 60 years (34.0 (IQR 18.0-49.0) or more than 60 years (36.0 (IQR 23.0-36.0) (p<0.019).  **Convergent validity:** KDQOL symptom/problem (r =-0.697), effects of kidney disease (r = -0.579), and burden (r=-0.512) negatively correlated (p<0.001) with Symptoms of CKD score. | **Test-retest reliability:** Reproducibility was high with a Spearman’s r value of >0.9. |
| FACT-An (Population: 145 anaemic patients with stage 3-4 CKD and 59 newly-initiated dialysis patients) ^43^ | **Convergent validity:** The FACT-Fatigue (r=0.76, p<0.001) and Anemia subscale (r=0.77, p<0.001) strongly correlated with the SF-36 vitality subscale. The FACT-An scores were modestly corelated at baseline due to the inclusion criteria. Correlations between FACT-An Total, Fatigue and AnS scores were higher (r=>0.30) at weeks 13/17.  **Known groups validity:** All key domains of FACT-An and SF-36 had highly significant differences: the FACT-An scores split by the SF-36 Physical Functioning domain were: FACT-Anemia subscale score (mean 46.4, [SD 13.9]) vs 61.6 (12.2), the FACT Fatigue subscale 28.9 (10.8) vs. 39.7 (9.5), and the Total FACT-An score 118.3 (28.8) vs 145.0 (25.0), all p < 0.0001.  **Responsiveness:** The FACT-An total and subscale scores except for social-wellbeing, indicated large improvements at week 9 and relatively stable by week 13/17 in Hb level. The mean scores were higher for the non-dialysis group compared to the dialysis group for FACT-An total score and all subscale scores. For example, the fatigue subscale: baseline: 0.11(p<0.05); week 9: 0.26(p<0.001); week 17: 0.30 (p<0.001). | **Internal consistency:** Good to excellent Cronbach’s α reliability coefficients (ranging from 0.79 to 0.95) was demonstrated for FACT-An total scores and subscales. A Cronbach’s α reliability coefficients of 0.93 was reported for FACT-An fatigue.  **Test-retest reliability:** The FACT-An fatigue subscale demonstrated good re-test reliability with an intra correlation coefficient of 0.80 with >0.6 intraclass correlation coefficient as an acceptable cut off^43^. Additionally, all total score and subscales demonstrated re-test reliability ranging from 0.72 to 0.88 |
| IPQ-R ^99^ | **Content validity:** Six out of 31 participants agreed with their full scores when interviewed 1 week to 2 months after IPQ-R data  were collected, the remaining 25 disagreed with at least one subscale. Average agreement rate = 79% (median = 86%, 29-100%). Subscale agreement rates: Timeline: 100%; Consequences, Cohernce and Emotion: 83%; Cyclical: 83%; Personal control: 65%; and Treatment control: 64%. Qualitative analysis found two main themes inaccuracy (31 of 43 disagreement responses) and uncertainty (12 of 43 disagreement responses). | **Internal consistency:** Cronbach’s alpha was determined moderate to high for all Illness Perceptions items: Timeline: 0.90; Cyclical: 0.84; Consequences: 0.77; Treatment control: 0.66; Personal control: 0.73; Illness coherence: 0.86; Emotion: representations: 0.86. |
| KSQ ^100^ | **Content validity:** No item received a poor relevance rating however ‘poor concentration’ had only fair validity. All scores fell within the recommended threshold of 0.80 with an the average I-CVI scores (S-CVI) for the KSQ as a whole 0.81.  **Convergent validity:** The frequency of 10 out of 13 items (itching, difficulty sleeping, loss of appetite, feeling tired, pain in bones or joints, poor concentration, loss of muscle strength, shortness of breath, muscle cramps or stiffness and feeling cold) were negatively associated with the EQ-5D-5L. The remaining three (lack of sex drive, need to urine, and back pain) were associated with the EQ-5D-5L index score at p<0.05 (though did not reach statistical significance under the Bonferroni correction). The EQ-5D index score and the frequency of pain in bones and joints observed the strongest relationship (rho=-0.709; p<0.001). | NA |
| SF-6D ^101^ (Population: 10% PD, 54% HD, 36% conservative care) | **Convergent validity:** The SF-6D Vitality domain was weakly correlated with the ICECAP-O Role (r=0.41, p<0.001) and Control (r=0.42, p<0.001) domains. | NA |
| SF-36 ^43^ (Population: (Non-dialysis = 145, dialysis = 59) | **Convergent validity:** The SF-36 vitality domain and FACT-Fatigue (r=0.76, p<0.001) and Anemia (r=0.77, p<0.001) subscales were strongly correlated.  **Known groups validity:** All FACT-An and SF-36 key domains reported highly significant differences. The SF-36 scores split  by the median FACT-An score showed large and significant differences (p < 0.0001) for Physical Functioning 32.3 (9.5) vs 43.8 (9.4); and Vitality 39.5 (13.5) vs. 55.5 (8.3).  **Responsiveness:** Small improvements were seen for SF-36 domains and component scores (relative to baseline). In the pre-dialysis group, only the vitality domain saw an increase greater than three-points by week 9 (p<0.001) or 17 (p<0.01).  For SF-36 domains and component scores, small improvements were seen (relative to baseline). For example, social functioning: baseline: 0.10 (p<0.05); week 9: 0.12 (p<0.001); week 17: 0.31 (p<0.001). However larger changes were seen in the dialysis group, only in the vitality domain saw an increase greater than three-points by week 9 (p<0.001) or 17 (p<0.01). | **Internal consistency:** Good to excellent Cronbach’s α reliability coefficients were demonstrated in the SF-36 domains and component summary score (ranging from 0.76-0.93) except for the General Health Domain (0.69). In particular, the Vitality domain with Cronbach’s α 0.82.  **Test-retest reliability:** All domains and summary scores demonstrated re-test reliability (ranging from 0.64 to 0.83) with >0.6 intraclass correlation coefficient as an acceptable cut off^43^. The Vitality domain reported an intraclass correlation coefficient of 0.64. |

CKD-SI: Chronic Kidney Disease Symptom Index) Sri Lanka Version; FACT-An: Functional Assessment of Cancer Therapy – Anemia; IPQ-R: Revised Illness Perception Questionnaire; KSQ: Kidney Symptom Questionnaire; SF-6D: Short-Form 6 Dimension; SF-36: 36-Item Short Form Health Survey: NA: Not available; MID: minimally important difference.

**References**

1. Akizawa T, Gejyo F, Nishi S, et al. Positive Outcomes of High Hemoglobin Target in Patients With Chronic Kidney Disease Not on Dialysis: A Randomized Controlled Study. *Therapeutic Apheresis and Dialysis.* 2011;15(5):431-440.

2. Alexander M, Kewalramani R, Agodoa I, Globe D. Association of anemia correction with health related quality of life in patients not on dialysis. *Curr Med Res Opin.* 2007;23(12):2997-3008.

3. Blakeman T, Blickem C, Kennedy A, et al. Effect of information and telephone-guided access to community support for people with chronic kidney disease: Randomised controlled trial. *PLoS ONE.* 2014;9(10) (no pagination).

4. Bohlke M, Barcellos FC, Santos IS, Mielke GI, de Marmann Vargas M, Hallal PC. Effects of a 16-week physical training on clinical outcomes in patients with hypertension and chronic kidney disease: NEPHROS post-trial follow-up. *Cadernos de Saude Publica.* 2022;38(5) (no pagination).

5. Campbell KL, Ash S, Bauer JD. The impact of nutrition intervention on quality of life in pre-dialysis chronic kidney disease patients. *Clinical Nutrition.* 2008;27(4):537-544.

6. Greenwood SA, Beckley-Hoelscher N, Asgari E, et al. The effect of intravenous iron supplementation on exercise capacity in iron-deficient but not anaemic patients with chronic kidney disease: study design and baseline data for a multicentre prospective double-blind randomised controlled trial. *BMC Nephrology.* 2022;23(1) (no pagination).

7. Hedayati SS, Gregg LP, Carmody T, et al. Effect of sertraline on depressive symptoms in patients with chronic kidney disease without dialysis dependence: The CAST randomized clinical trial. *JAMA - Journal of the American Medical Association.* 2017;318(19):1876-1890.

8. Hirakata H, Tsubakihara Y, Gejyo F, et al. Maintaining high hemoglobin levels improved the left ventricular mass index and quality of life scores in pre-dialysis Japanese chronic kidney disease patients. *Clin Exp Nephrol.* 2010;14(1):28-35.

9. Lewis EF, Pfeffer MA, Feng A, et al. Darbepoetin alfa impact on health status in diabetes patients with kidney disease: a randomized trial. *Clinical Journal of The American Society of Nephrology: CJASN.* 2011;6(4):845-855.

10. Li WY, Chiu FC, Zeng JK, et al. Mobile health app with social media to support self-management for patients with chronic kidney disease: Prospective randomized controlled study. *Journal of Medical Internet Research.* 2020;22(12) (no pagination).

11. Mager DR, Jackson ST, Hoffmann MR, Jindal K, Senior PA. Vitamin D<inf>3</inf> supplementation, bone health and quality of life in adults with diabetes and chronic kidney disease: Results of an open label randomized clinical trial. *Clinical Nutrition.* 2017;36(3):686-696.

12. Mustata S, Groeneveld S, Davidson W, Ford G, Kiland K, Manns B. Effects of exercise training on physical impairment, arterial stiffness and health-related quality of life in patients with chronic kidney disease: A pilot study. *International Urology and Nephrology.* 2011;43(4):1133-1141.

13. Provenzano R, Bhaduri S, Singh AK, Group PS. Extended epoetin alfa dosing as maintenance treatment for the anemia of chronic kidney disease: the PROMPT study. *Clinical Nephrology.* 2005;64(2):113-123.

14. Roger SD, Jassal SV, Woodward MC, Soroka S, McMahon LP. A randomised single-blind study to improve health-related quality of life by treating anaemia of chronic kidney disease with Aranesp R (darbepoetin alfa) in older people: STIMULATE. *Int Urol Nephrol.* 2014;46(2):469-475.

15. Rossi AP, Burris DD, Lucas FL, Crocker GA, Wasserman JC. Effects of a renal rehabilitation exercise program in patients with CKD: a randomized, controlled trial. *Clinical Journal of The American Society of Nephrology: CJASN.* 2014;9(12):2052-2058.

16. Shin J, Hwang JH, Han M, et al. Phase angle as a marker for muscle health and quality of life in patients with chronic kidney disease. *Clinical Nutrition.* 2022;41(8):1651-1659.

17. Uchiyama K, Adachi K, Muraoka K, et al. Home-based aerobic exercise and resistance training for severe chronic kidney disease: a randomized controlled trial. *J Cachexia Sarcopenia Muscle.* 2021;12(6):1789-1802.

18. Van Craenenbroeck AH, Van Craenenbroeck EM, Van Ackeren K, et al. Effect of Moderate Aerobic Exercise Training on Endothelial Function and Arterial Stiffness in CKD Stages 3-4: A Randomized Controlled Trial. *American Journal of Kidney Diseases.* 2015;66(2):285-296.

19. Villar E, Lievre M, Kessler M, et al. Anemia normalization in patients with type 2 diabetes and chronic kidney disease: Results of the NEPHRODIAB2 randomized trial. *Journal of Diabetes and its Complications.* 2011;25(4):237-243.

20. Yu-Hsiu K, Yi-Ching H, Pei-Ying C, Kuo-Ming W. The effects of exercise education intervention on the exercise behaviour, depression, and fatigue status of chronic kidney disease patients. *Health Education (0965-4283).* 2012;112(6):473-484.

21. Guo Y, Zhang M, Ye T, et al. Non-protein energy supplement for malnutrition treatment in patients with chronic kidney disease. *Asia Pacific journal of clinical nutrition.* 2022;31(3):504-511.

22. Lefebvre P, Vekeman F, Sarokhan B, Enny C, Provenzano R, Cremieux PY. Relationship between hemoglobin level and quality of life in anemic patients with chronic kidney disease receiving epoetin alfa. *Current Medical Research and Opinion.* 2006;22(10):1929-1937.

23. Abeywickrama HM, Wimalasiri S, Koyama Y, et al. Quality of life and symptom burden among chronic kidney disease of uncertain etiology (CKDU) patients in Girandurukotte, Sri Lanka. *International Journal of Environmental Research and Public Health.* 2020;17(11):1-16.

24. Adame Perez SI, Senior PA, Field CJ, Jindal K, Mager DR. Frailty, Health-Related Quality of Life, Cognition, Depression, Vitamin D and Health-Care Utilization in an Ambulatory Adult Population With Type 1 or Type 2 Diabetes Mellitus and Chronic Kidney Disease: A Cross-Sectional Analysis. *Canadian Journal of Diabetes.* 2019;43(2):90-97.

25. Aggarwal HK, Jain D, Pawar S, Yadav RK. Health-related quality of life in different stages of chronic kidney disease. *Qjm.* 2016;109(11):711-716.

26. Arnold R, Pianta TJ, Issar T, et al. Peripheral neuropathy: An important contributor to physical limitation and morbidity in stages 3 and 4 chronic kidney disease. *Nephrology Dialysis Transplantation.* 2022;37(4):713-719.

27. Bagasha P, Namukwaya E, Leng M, et al. Comparison of the health-related quality of life of end stage kidney disease patients on hemodialysis and non-hemodialysis management in Uganda. *BMC Palliat Care.* 2021;20(1):52.

28. Bonner A, Caltabiano M, Berlund L. Quality of life, fatigue, and activity in Australians with chronic kidney disease: a longitudinal study. *Nursing & health sciences.* 2013;15(3):360-367.

29. Brown SA, Tyrer FC, Clarke AL, et al. Symptom burden in patients with chronic kidney disease not requiring renal replacement therapy. *Clinical Kidney Journal.* 2017;10(6):788-796.

30. Canales MT, Bozorgmehri S, Ishani A, Weiner I, Berry R, Beyth R. Prevalence and correlates of sleep apnea among US veterans with chronic kidney disease. *Journal of Sleep Research Vol 29(4), 2020, ArtID e12981.* 2020;29(4).

31. Chan GCW, Lam B, Yap DYH, Ip MSM, Lai KN, Tang SCW. Proteinuria is associated with sleep apnea in chronic kidney disease. *Nephrology Dialysis Transplantation.* 2016;31(5):772-779.

32. Chesnaye NC, Meuleman Y, de Rooij ENM, et al. Health-Related Quality-of-Life Trajectories over Time in Older Men and Women with Advanced Chronic Kidney Disease. *Clinical Journal of the American Society of Nephrology.* 2022;17(2):205-214.

33. Chin HJ, Song YR, Lee JJ, et al. Moderately decreased renal function negatively affects the health-related quality of life among the elderly Korean population: A population-based study. *Nephrology Dialysis Transplantation.* 2008;23(9):2810-2817.

34. Davison SN, Jhangri GS, Feeny DH. Comparing the Health Utilities Index Mark 3 (HUI3) with the Short Form-36 preference-based SF-6D in chronic kidney disease. *Value in Health.* 2009;12(2):340-345.

35. de Goeij MC, Ocak G, Rotmans JI, Eijgenraam JW, Dekker FW, Halbesma N. Course of symptoms and health-related quality of life during specialized pre-dialysis care. *PLoS ONE [Electronic Resource].* 2014;9(4):e93069.

36. Delgado CE, Jaramillo MM, Orozco BE, et al. [Quality of life in patients with chronic kidney disease without dialysis or transplant: a random sample from two insurance companies. Medellin, Colombia, 2008]. *Nefrologia.* 2009;29(6):548-556.

37. Eltawdy M, Rabah A, Nada M, Refaat R, Afifi L. Sleep disorders in chronic kidney disease patients. *Egyptian Journal of Neurology, Psychiatry and Neurosurgery.* 2016;53(1):48-53.

38. Erez G, Selman L, Murtagh FE. Measuring health-related quality of life in patients with conservatively managed stage 5 chronic kidney disease: limitations of the Medical Outcomes Study Short Form 36: SF-36. *Quality of Life Research.* 2016;25(11):2799-2809.

39. Farag YM, Keithi-Reddy SR, Mittal BV, et al. Anemia, inflammation and health-related quality of life in chronic kidney disease patients. *Clinical Nephrology.* 2011;75(6):524-533.

40. Faye M, Legrand K, Le Gall L, et al. Five-Year Symptom Trajectories in Nondialysis-Dependent CKD Patients. *Clinical Journal of the American Society of Nephrology.* 2022;17(11):1588-1597.

41. Feng L, Yap KB, Ng TP. Depressive symptoms in older adults with chronic kidney disease: mortality, quality of life outcomes, and correlates. *Am J Geriatr Psychiatry.* 2013;21(6):570-579.

42. Finkelstein FO, Story K, Firanek C, et al. Health-related quality of life and hemoglobin levels in chronic kidney disease patients. *Clinical Journal of the American Society of Nephrology.* 2009;4(1):33-38.

43. Finkelstein FO, van Nooten F, Wiklund I, Trundell D, Cella D. Measurement properties of the Short Form-36 (SF-36) and the Functional Assessment of Cancer Therapy - Anemia (FACT-An) in patients with anemia associated with chronic kidney disease. *Health and Quality of Life Outcomes.* 2018;16(1):111.

44. Gregg LP, Jain N, Carmody T, et al. Fatigue in nondialysis chronic kidney disease: Correlates and association with kidney outcomes. *American Journal of Nephrology.* 2019;50(1):37-47.

45. Guedes M, Muenz D, Zee J, et al. Serum biomarkers of iron stores are associated with worse physical health-related quality of life (HRQoL) in non-dialysis dependent chronic kidney disease (NDD-CKD) patients with or without anemia. *Nephrology, dialysis, transplantation : official publication of the European Dialysis and Transplant Association European Renal Association.* 2021;24.

46. Gunawardena N, Palihawadana P, Senanayake S, Karunarathna R, Kumara P, Kularatna S. Health related quality of life in chronic kidney disease; A descriptive study in a rural Sri Lankan community affected by chronic kidney disease. *Health and Quality of Life Outcomes.* 2020;18(1) (no pagination).

47. Gyamlani G, Basu A, Geraci S, et al. Depression, screening and quality of life in chronic kidney disease. *American Journal of the Medical Sciences.* 2011;342(3):186-191.

48. Hakamaki M, Lankinen R, Hellman T, et al. Quality of Life Is Associated with Cardiac Biomarkers, Echocardiographic Indices, and Mortality in CKD Stage 4-5 Patients Not on Dialysis. *Blood Purification.* 2021;50(3):347-354.

49. Hamilton R, Hawley S. Quality of life outcomes related to anemia management of patients with chronic renal failure. *Clinical nurse specialist CNS.* 2006;20(3):139-143; quiz 144-145.

50. Hanson CS, Gutman T, Craig JC, et al. Identifying Important Outcomes for Young People With CKD and Their Caregivers: A Nominal Group Technique Study. *American Journal of Kidney Diseases.* 2019;74(1):82-94.

51. Hao CM, Wittbrodt ET, Palaka E, Guzman N, Dunn A, Grandy S. Understanding patient perspectives and awareness of the impact and treatment of anemia with chronic kidney disease: A patient survey in China. *International Journal of Nephrology and Renovascular Disease.* 2021;14:53-64.

52. Ho YF, Hsu PT, Yang KL. The mediating effect of sleep quality and fatigue between depression and renal function in nondialysis chronic kidney disease: a cross-sectional study. *BMC Nephrol.* 2022;23(1):126.

53. Hoshino J, Muenz D, Zee J, et al. Associations of Hemoglobin Levels With Health-Related Quality of Life, Physical Activity, and Clinical Outcomes in Persons With Stage 3-5 Nondialysis CKD. *J Ren Nutr.* 2020;30(5):404-414.

54. Iliescu EA, Yeates KE, Holland DC. Quality of sleep in patients with chronic kidney disease. *Nephrology Dialysis Transplantation.* 2004;19(1):95-99.

55. Jalal SM, Beth MRM, Bo Khamseen ZM. Impact of Hospitalization on the Quality of Life of Patients with Chronic Kidney Disease in Saudi Arabia. *International Journal of Environmental Research & Public Health [Electronic Resource].* 2022;19(15):07.

56. Jhamb M, Liang K, Yabes J, et al. Prevalence and correlates of fatigue in chronic kidney disease and end-stage renal disease: Are sleep disorders a key to understanding fatigue? *American Journal of Nephrology.* 2014;38(6):489-495.

57. Kaltsouda A, Skapinakis P, Damigos D, et al. Defensive coping and health-related quality of life in chronic kidney disease: a cross-sectional study. *BMC Nephrology.* 2011;12:28.

58. Kefale B, Alebachew M, Tadesse Y, Engidawork E. Quality of life and its predictors among patients with chronic kidney disease: A hospital-based cross sectional study. *PLoS ONE [Electronic Resource].* 2019;14(2):e0212184.

59. Kharshid AM, Sulaiman SAS, Saadh MJ. Health-related quality of life in chronic kidney disease patients: A cross-sectional study. *Systematic Reviews in Pharmacy.* 2020;11(7):188-192.

60. Kim HJ, Kim DW, Rhee H, et al. Rapid decline in kidney function is associated with rapid deterioration of health-related quality of life in chronic kidney disease. *Scientific reports.* 2023;13(1):1786.

61. Knutson KL, Lash J, Ricardo AC, et al. Habitual sleep and kidney function in chronic kidney disease: the Chronic Renal Insufficiency Cohort study. *J Sleep Res.* 2018;27(2):281-289.

62. Korevaar JC, Jansen MA, Merkus MP, Dekker FW, Boeschoten EW, Krediet RT. Quality of life in predialysis end-stage renal disease patients at the initiation of dialysis therapy. The NECOSAD Study Group. *Perit Dial Int.* 2000;20(1):69-75.

63. Krishnasamy R, Hawley CM, Stanton T, et al. Association between left ventricular global longitudinal strain, health-related quality of life and functional capacity in chronic kidney disease patients with preserved ejection fraction. *Nephrology.* 2016;21(2):108-115.

64. Kularatna S, Senanayake S, Gunawardena N, Graves N. Comparison of the EQ-5D 3L and the SF-6D (SF-36) contemporaneous utility scores in patients with chronic kidney disease in Sri Lanka: A Cross-sectional survey. *BMJ Open.* 2019;9(2) (no pagination).

65. Kumar B, Tilea A, Gillespie BW, et al. Significance of self-reported sleep quality (SQ) in chronic kidney disease (CKD): The Renal Research Institute (RRI)-CKD study. *Clinical Nephrology.* 2010;73(2):104-114.

66. Kusek JW, Greene P, Wang SR, et al. Cross-sectional study of health-related quality of life in African Americans with chronic renal insufficiency: The African American Study of Kidney Disease and Hypertension Trial. *American Journal of Kidney Diseases.* 2002;39(3):513-524.

67. Lemos CF, Rodrigues MP, Veiga JR. Family income is associated with quality of life in patients with chronic kidney disease in the pre-dialysis phase: a cross sectional study. *Health & Quality of Life Outcomes.* 2015;13:202.

68. Lin SF, Fan YC, Kuo TT, Pan WH, Bai CH. Quality of life and cognitive assessment in healthy older Asian people with early and moderate chronic kidney disease: The NAHSIT 2013-2016 and validation study. *PLoS ONE.* 2022;17(3 March) (no pagination).

69. MacDonald JH, Fearn L, Jibani M, Marcora SM. Exertional fatigue in patients with CKD. *American Journal of Kidney Diseases.* 2012;60(6):930-939.

70. Mansur HN, Colugnati FA, Grincenkov FR, Bastos MG. Frailty and quality of life: a cross-sectional study of Brazilian patients with pre-dialysis chronic kidney disease. *Health & Quality of Life Outcomes.* 2014;12:27.

71. Martini A, Ammirati A, Garcia C, et al. Evaluation of quality of life, physical, and mental aspects in longevous patients with chronic kidney disease. *International Urology and Nephrology.* 2018;50(4):725-731.

72. McKercher CM, Venn AJ, Blizzard L, et al. Psychosocial factors in adults with chronic kidney disease: Characteristics of pilot participants in the Tasmanian Chronic Kidney Disease study. *BMC Nephrology.* 2013;14(1) (no pagination).

73. Mujais SK, Story K, Brouillette J, et al. Health-related quality of Life in CKD patients: Correlates and evolution over time. *Clinical Journal of the American Society of Nephrology.* 2009;4(8):1293-1301.

74. Nicholl DDM, Ahmed SB, Loewen AHS, et al. Clinical presentation of obstructive sleep apnea in patients with chronic kidney disease. *Journal of Clinical Sleep Medicine.* 2012;8(4):381-387.

75. Nixon AC, Wilkinson TJ, Young HML, et al. Symptom-burden in people living with frailty and chronic kidney disease. *BMC Nephrology.* 2020;21(1) (no pagination).

76. Oh TR, Choi HS, Kim CS, et al. Association between health related quality of life and progression of chronic kidney disease. *Scientific Reports.* 2019;9(1):19595.

77. Oh TR, Choi HS, Suh SH, et al. The Association between Health-Enhancing Physical Activity and Quality of Life in Patients with Chronic Kidney Disease: Propensity Score Matching Analysis. *International Journal of Environmental Research and Public Health.* 2022;19(3) (no pagination).

78. Oh TR, Kim CS, Bae EH, et al. Association between vitamin D deficiency and health-related quality of life in patients with chronic kidney disease from the KNOW-CKD study. *PLoS ONE [Electronic Resource].* 2017;12(4):e0174282.

79. Okoro RN, Adibe MO, Okonta MJ, Ummate I, Ohieku JD, Yakubu S. Assessment of health-related quality of life and its determinants in the pre-dialysis patients with chronic kidney disease. *Int Urol Nephrol.* 2022;54(1):165-172.

80. Peng T, Hu Z, Guo L, Xia Q, Li D, Yang X. Relationship between psychiatric disorders and quality of life in nondialysis patients with chronic kidney disease. *American Journal of the Medical Sciences.* 2013;345(3):218-221.

81. Pereira RA, Alvarenga MDS, de Andrade LS, et al. Effect of a nutritional behavioral intervention on intuitive eating in overweight women with chronic kidney disease. *Journal of renal nutrition : the official journal of the Council on Renal Nutrition of the National Kidney Foundation.* 2022;02.

82. Perlman RL, Finkelstein FO, Liu L, et al. Quality of life in Chronic Kidney Disease (CKD): A cross-sectional analysis in the Renal Research Institute-CKD study. *American Journal of Kidney Diseases.* 2005;45(4):658-666.

83. Picard K, Senior PA, Adame Perez S, Jindal K, Richard C, Mager DR. Low Mediterranean Diet scores are associated with reduced kidney function and health related quality of life but not other markers of cardiovascular risk in adults with diabetes and chronic kidney disease. *Nutrition, Metabolism and Cardiovascular Diseases.* 2021;31(5):1445-1453.

84. Rajan M, Lai KC, Tseng CL, et al. Estimating utilities for chronic kidney disease, using SF-36 and SF-12-based measures: challenges in a population of veterans with diabetes. *Quality of life research : an international journal of quality of life aspects of treatment, care and rehabilitation.* 2013;22(1):53-64.

85. Ruszkowski J, Heleniak Z, Krol E, Tarasewicz A, Witkowski JM, Debska-Slizien A. Associations between constipation symptoms and the sleep quality in non-dialysis chronic kidney disease patients: a cross-sectional study. *Polish archives of internal medicine.* 2021;27.

86. Seidel UK, Gronewold J, Volsek M, et al. Physical, cognitive and emotional factors contributing to quality of life, functional health and participation in community dwelling in chronic kidney disease. *PLoS ONE.* 2014;9(3) (no pagination).

87. Senanayake S, Gunawardena N, Palihawadana P, et al. Health related quality of life in chronic kidney disease; a descriptive study in a rural Sri Lankan community affected by chronic kidney disease. *Health & Quality of Life Outcomes.* 2020;18(1):106.

88. Tannor EK, Norman BR, Adusei KK, Sarfo FS, Davids MR, Bedu-Addo G. Quality of life among patients with moderate to advanced chronic kidney disease in Ghana - A single centre study. *BMC Nephrology.* 2019;20(1) (no pagination).

89. Taptagaporn S, Mongkolsomlit S, Rakkapao N, Kaewdok T, Wattanasoei S. Quality of life among patients suffering from chronic kidney disease in chronic kidney disease clinic of thailand. *Open Public Health Journal.* 2021;14(1):417-424.

90. Tesfaye WH, McKercher C, Peterson GM, et al. Medication adherence, burden and health-related quality of life in adults with predialysis chronic kidney disease: A prospective cohort study. *International Journal of Environmental Research and Public Health.* 2020;17(1) (no pagination).

91. Wan Zukiman WZH, Yaakup H, Zakaria NF, Shah SAB. Symptom Prevalence and the Negative Emotional States in End-Stage Renal Disease Patients with or without Renal Replacement Therapy: A Cross-Sectional Analysis. *J Palliat Med.* 2017;20(10):1127-1134.

92. Wang WL, Liang S, Zhu FL, et al. The prevalence of depression and the association between depression and kidney function and health-related quality of life in elderly patients with chronic kidney disease: A multicenter cross-sectional study. *Clinical Interventions in Aging.* 2019;14:905-913.

93. Wee HL, Seng BJ, Lee JJ, et al. Association of anemia and mineral and bone disorder with health-related quality of life in Asian pre-dialysis patients. *Health & Quality of Life Outcomes.* 2016;14:94.

94. Wirkner J, Scheuch M, Dabers T, et al. Comorbid Depression and Diabetes Are Associated with Impaired Health-Related Quality of Life in Chronic Kidney Disease Patients. *Journal of Clinical Medicine.* 2022;11(16) (no pagination).

95. Wulczyn KE, Zhao SH, Rhee EP, Kalim S, Shafi T. Trajectories of Uremic Symptom Severity and Kidney Function in Patients with Chronic Kidney Disease. *Clinical Journal of the American Society of Nephrology.* 2022;17(4):496-506.

96. Xiong J, Peng H, Yu Z, et al. Daily Walking Dose and Health-related Quality of Life in Patients With Chronic Kidney Disease. *Journal of renal nutrition : the official journal of the Council on Renal Nutrition of the National Kidney Foundation.* 2022;32(6):710-717.

97. Yapa HE, Purtell L, Chambers S, Bonner A. Alterations in symptoms and health-related quality of life as kidney function deteriorates: A cross-sectional study. *J Clin Nurs.* 2021;30(11-12):1787-1796.

98. Senanayake SJ GN, Palihawadana P. Development of the Chronic Kidney Disease Symptom Index – Sri Lanka; a symptom assessment instrument for Chronic Kidney Disease patients. *Journal of the Postgraduate Institute of Medicine.* 2017;4(1):E38 31-12.

99. Rivera E, Levoy K, Clark-Cutaia MN, et al. Content Validity Assessment of the Revised Illness Perception Questionnaire in CKD Using Qualitative Methods. *Int J Environ Res Public Health.* 2022;19(14).

100. Brown SA, Tyrer F, Clarke AL, et al. Kidney symptom questionnaire: Development, content validation and relationship with quality of life. *Journal of Renal Care.* 2018;44(3):162-173.

101. Karan KS, Fliss EMM, Kevin M, et al. Health-related quality of life and well-being in people over 75 years of age with end-stage kidney disease managed with dialysis or comprehensive conservative care: a cross-sectional study in the UK and Australia. *BMJ Open.* 2019;9(5):e027776.
