# Supplementary material for: Patient-reported outcome measures for fatigue in patients with chronic kidney disease: a systematic review
Source: BMJ Open. 2025 Jul 16;15(7):e099592. doi: 10.1136/bmjopen-2025-099592 (PMC12273140; doi:10.1136/bmjopen-2025-099592)
Supplement: Supplementary file 2 [file bmjopen-2025-099592.reviewer_comments.pdf]

PEER REVIEW HISTORY

BMJ Open publishes all reviews undertaken for accepted manuscripts. Reviewers are asked to complete a checklist review form and are provided with free text boxes to elaborate on their assessment. These free text comments are reproduced below.

ARTICLE DETAILS

Title (Provisional)

Patient-reported outcome measures for fatigue in patients with chronic kidney disease: a systematic review

Authors

Hughes, Anastasia; Ju, Angela; Cazzolli, Rosanna; Howell, Martin; Guha, Chandana; Levin, Adeera; Manera , Karine; Teixeira-Pinto, Armando; Torrisi, Luca G; Wheeler, David; Wong, Germaine; Wu, Rebecca; Jaure, Allison

---

VERSION 1 - REVIEW

**Reviewer**	**1**
**Name**	**Shaheen, Naila**
**Affiliation**	**King Saud bin Abdulaziz University for Health Sciences, Biostatistics**
**Date**	**03-Mar-2025**
**COI**	**None to be declared**

Well-done! A very nice work and well-written paper. The paper is tackling an important research question.

Minor modifications are suggested for improvement purposes.

Though as a broader category it is termed as measures, however I think replacing measure with tool is more appropriate throughout the paper where ever is applicable, as usually we call it as a validated tool as used on page 10; line 38

"However, the tools do provide important insights and potential measures to be put forward when designing and/or validating a new measure."

Suggest modifying measures/ tools

Page 7; line 5 measures/ tools

Table a,b,c measure/ tools

Methods:

Page 6; line 7-11: How the content was derived? Based on the existing PROMs?

Results:

- Fig 1: Is it citations? Usually it is the number of articles. Please delete citations. Can add n e.g. EMBASE n=3459

- Table 1: replace past month to past 4 weeks.

- Content of Measures: Please add referring to which figure/ table

- Table c: Delete column intervention

Discussion:

- Page 10; line 11 Please replace analysis with results. Our results indicate…..

**Reviewer**	**2**
**Name**	**Kyte, Derek**
**Affiliation**	**University of Worcester**
**Date**	**27-Mar-2025**
**COI**	**In the last 5 years I have received funding from the NIHR and Kidney Research UK (KRUK).**

KRUK funding has supported my work to develop a disease-specific kidney symptom burden questionnaire (Stoneygate Research Award, KS_RP_013_20180914).

Introduction

The focus on fatigue is well justified. Fatigue is well recognised as one of the most prevalent symptoms affecting people with CKD globally.

The rationale for the study was well presented and the aims clear.

Methods

Did the authors adhere to any existing guidance when performing their review (e.g., PRISMA, COSMIN)? This should be mentioned at the start of the methods section and links added to any appropriate checklists made available in the appendices. Note: Coming back to this comment now that I have read the whole paper, I can see that the PRISMA checklist appears in the appendix, this obviously needs to be made clearer in the methods. In addition, I do find it surprising that the COSMIN guidelines for systematic reviews of patient-reported outcome measures were not mentioned.

Can the authors confirm that a single author was involved in the searching, screening and data extraction processes -as this is how it comes across in the wording of the manuscript? If this is the case, it raises the risk of errors and may impact the credibility of the study and its findings.

Did the authors find evidence to support their assumption of 12 seconds per item, where no completion time data were available for a particular measure? If so, this should made clearer. Equally, if there was no underpinning evidence, this should be clarified.

The exact search strategy for the validation studies was not clear. I did not see details of this in the supplementary appendix. Was the COSMIN search filter used for this? This needs clarifying.

Detail around the exact process for assessing the psychometric properties of the outcome measures was limited. A summary of the process would be useful. In addition, whilst the table in the supplementary appendix does outline the measurement properties of interest, there is no detail for the reader of the minimum requirements for each property. This may make interpretation of the findings of this review challenging.

It was not clear until the 'Public and patient involvement in research' section that the project was linked to the SONG initiative. It may be useful to signpost this earlier in the manuscript.

Results

Page 8, Line 18/19, reference missing the year: 'Chen & Ku, and Hao et al'

It did not appear that all PROMs were named in full within the main manuscript before acronyms were presented, the authors should double check this throughout.

I found that whilst most tables were informative, Table 5 could be improved. It currently simply highlights whether a measurement property had been assessed (or not) for a given measure. However, it could also provide evidence regarding the findings of the assessment. I feel that is important that this information is incorporated (as it is in many COSMIN reviews).

Discussion

The authors state: "However, it is reported infrequently in trials and observational studies in patients with CKD." It would be useful to provide data on the proportions, e.g. 2 RCTs were found to report fatigue, but out of how many trials in total that were screened during the review which included patients with CKD not requiring KRT?

Again, reference is made to SONG initiatives, this makes it even more important to signpost the relationship at the start of the manuscript.

Overall

The manuscript is well written and presents important findings. The authors should be congratulated on their work.

VERSION 1 - AUTHOR RESPONSE

Reviewer 1: Dr. Naila Shaheen, King Saud bin Abdulaziz University for Health Sciences.

1. **Though as a broader category it is termed as measures, however I think replacing measure with tool is more appropriate throughout the paper where ever is applicable, as usually we call it as a validated tool as used on page 10; line 38 "However, the tools do provide important insights and potential measures to be put forward when designing and/or validating a new measure." Suggest modifying measures/ tools. Page 7; line 5 measures/ tools. Table a,b,c measure/ tools.**
   As suggested, we have updated the term to "tool" where appropriate and applicable.

2. **Methods: Page 6; line 7-11: How the content was derived? Based on the existing PROMs?**
   The dimensions of fatigue were derived based on a recent systematic review of PROMs for fatigue in patients with kidney failure receiving haemodialysis. We have now clarified: *"The dimensions of fatigue was derived from a systematic review of PROMs for fatigue in dialysis[1]. To ensure all dimensions were included, we also examined the Patient-Reported Outcomes Measurement Information System (PROMIS) fatigue measure, no additional dimensions were identified[2]"* (page 6, paragraph 1 – marked copy).

3. **Results: Fig 1: Is it citations? Usually, it is the number of articles. Please delete citations. Can add n e.g. EMBASE n=3459**
   To clarify, the numbers in figure 1 refers the number of articles at each stage of screening. We have updated the figure to state n rather than citations.

4. **Table 1: replace past month to past 4 weeks.**
   We prefer to state the recall period from the original tool.

5. **Content of Measures: Please add referring to which figure/ table.**
   As suggested, we have now added a reference to the specific table displaying the content of measures: *"The content of measures is shown in tables 3 and 4"* (page 7, paragraph 4 – marked copy).

6. **Table c: Delete column intervention.**
   As suggested, we have removed the column of interventions.

7. **Discussion: Page 10; line 11 Please replace analysis with results. Our results indicate…..**
   We have now changed the phrase to state: "Our results indicate" (page 9, paragraph 4 – marked copy).

Reviewer 2: Dr. Derek Kyte, University of Worcester

8. **Methods: Did the authors adhere to any existing guidance when performing their review (e.g., PRISMA, COSMIN)? This should be mentioned at the start of the methods section and links added to any appropriate checklists made available in the appendices. Note: Coming back to this comment now that I have read the whole paper, I can see that the PRISMA checklist appears in the appendix, this obviously needs to be made clearer in the methods. In addition, I do find it surprising that the COSMIN guidelines for systematic reviews of patient-reported outcome measures were not mentioned.**
   We have now indicated that we followed the COSMIN guidelines for systematic review on patient-reported outcome measures. *"The systematic review adheres to the COSMIN guidelines for systematic reviews on patient-report outcome measures[3]"* (page 5, paragraph 1). We also confirm that we used PRISMA to report the steps of our systematic review where appropriate.

9. **Can the authors confirm that a single author was involved in the searching, screening and data extraction processes -as this is how it comes across in the wording of the manuscript? If this is the case, it raises the risk of errors and may impact the credibility of the study and its findings.**
   We have now added: *"The initial literature search and screening were conducted by author (AH) and search results were reviewed by author AJa to ensure accuracy."* (page 5, paragraph 2). *"The data extraction was conducted by AH and checked by author AJa."* (page x, paragraph y – marked copy)

10. **Did the authors find evidence to support their assumption of 12 seconds per item, where no completion time data were available for a particular measure? If so, this should made clearer. Equally, if there was no underpinning evidence, this should be clarified.**
    The estimation of 12 seconds per item is consistent with other systematic reviews of patient-reported outcomes, and the completion times of other measures. We have now added a reference to support this[4,5].

11. **The exact search strategy for the validation studies was not clear. I did not see details of this in the supplementary appendix. Was the COSMIN search filter used for this? This needs clarifying.**
    We have now clarified this adding the following sentence: *"As recommended by COSMIN-COMET we manually searched for the validation studies of measures identified in the review reporting psychometric robustness in patient with CKD not requiring kidney replacement therapy"* (page 6, paragraph 2).

12. **Detail around the exact process for assessing the psychometric properties of the outcome measures was limited. A summary of the process would be useful. In addition, whilst the table in the supplementary appendix does outline the measurement properties of interest, there is no detail for the reader of the minimum requirements for each property. This may make interpretation of the findings of this review challenging.**
    As suggested, we have elaborated on the process for assessing the psychometric properties of the tools: *"We searched for all validation studies reporting the psychometric robustness of the measure and extracted the psychometric assessment"* (page 6, paragraph 2).

13. **It was not clear until the 'Public and patient involvement in research' section that the project was linked to the SONG initiative. It may be useful to signpost this earlier in the manuscript.**
    As advised, we have added the following sentence to the introduction: *"Through the international Standardised Outcomes in Nephrology – Chronic Kidney Disease (SONG-CKD) Initiative, which involved 1400 patients, caregivers and health professionals from 73 countries, fatigue was identified as an important outcome to be reported in trials involving patients with CKD prior to the need for KRT[6,7]"* (marked copy – page 4, paragraph 2).

14. **Page 8, Line 18/19, reference missing the year: 'Chen & Ku, and Hao et al'.**
    We have now added the year to the reference.

15. **It did not appear that all PROMs were named in full within the main manuscript before acronyms were presented, the authors should double check this throughout.**
    We have now ensured that all PROMs are named in full.

16. **I found that whilst most tables were informative, Table 5 could be improved. It currently simply highlights whether a measurement property had been assessed (or not) for a given measure. However, it could also provide evidence regarding the findings of the assessment. I feel that is important that this information is incorporated (as it is in many COSMIN reviews).**
    Table 5 provides a summary of the measurement properties that have been assessed. We note (and referenced in the manuscript) that Supplementary File 4 provides a more detailed table that outlines the results from the validation studies on the psychometric properties of the tools.

17. **The authors state: "However, it is reported infrequently in trials and observational studies in patients with CKD." It would be useful to provide data on the proportions, e.g. 2 RCTs were found to report fatigue, but out of how many trials in total that were screened during the review which included patients with CKD not requiring KRT?**
    We confirm that all trials and observational studies included patients with CKD not requiring KRT and have now stated: *"patients with CKD not requiring kidney replacement therapy"* (marked copy – page 9, paragraph 4).

18. **Again, reference is made to SONG initiatives, this makes it even more important to signpost the relationship at the start of the manuscript.**
    Please see our response to Point #16 above.

References

1. Ju A, Unruh ML, Davison SN, et al. Patient-Reported Outcome Measures for Fatigue in Patients on Hemodialysis: A Systematic Review. *American Journal of Kidney Diseases.* 2018;71(3):327-343.
2. Cook KF, Jensen SE, Schalet BD, et al. PROMIS measures of pain, fatigue, negative affect, physical function, and social function demonstrated clinical validity across a range of chronic conditions. *Journal of Clinical Epidemiology.* 2016;73:89-102.
3. Elsman EBM, Mokkink LB, Terwee CB, et al. Guideline for reporting systematic reviews of outcome measurement instruments (OMIs): PRISMA-COSMIN for OMIs 2024. *Health and Quality of Life Outcomes.* 2024;22(1):48.
4. Manera KE, Ju A, Baumgart A, et al. Patient-reported outcome measures for life participation in peritoneal dialysis: a systematic review. *Nephrol Dial Transplant.* 2021;36(5):890-901.
5. Jacobson J, Ju A, Baumgart A, et al. Patient Perspectives on the Meaning and Impact of Fatigue in Hemodialysis: A Systematic Review and Thematic Analysis of Qualitative Studies. *American Journal of Kidney Diseases.* 2019;74(2):179-192.
6. Matus Gonzalez A, Evangelidis N, Howell M, et al. Outcomes for clinical trials involving adults with chronic kidney disease: a multinational Delphi survey involving patients, caregivers, and health professionals. *Nephrology Dialysis Transplantation.* 2024:gfae010.
7. González AM, Gutman T, Lopez-Vargas P, et al. Patient and Caregiver Priorities for Outcomes in CKD: A Multinational Nominal Group Technique Study. *American Journal of Kidney Diseases.* 2020;76(5):679-689.
